# Conformational plasticity in the KcsA potassium channel pore helix revealed by homo-FRET studies

**DOI:** 10.1038/s41598-019-42405-5

**Published:** 2019-04-17

**Authors:** M. Lourdes Renart, A. Marcela Giudici, José A. Poveda, Aleksander Fedorov, Mário N. Berberan-Santos, Manuel Prieto, Clara Díaz-García, José M. González-Ros, Ana Coutinho

**Affiliations:** 1 CQFM-IN and iBB–Institute for Bioengineering and Bioscience, Instituto Superior Técnico, Universidade de Lisboa, 1049-001 Lisboa, Portugal; 2 IBMC and IDiBE-Instituto de Investigación, Desarrollo e Innovación en Biotecnología Sanitaria de Elche, Universidad Miguel Hernández, Elche, 03202 Alicante Spain; 3 Departamento de Química e Bioquímica, Faculty of Sciences, Universidade de Lisboa, 1749-016 Lisboa, Portugal

**Keywords:** Biological fluorescence, Membrane proteins

## Abstract

Potassium channels selectivity filter (SF) conformation is modulated by several factors, including ion-protein and protein-protein interactions. Here, we investigate the SF dynamics of a single Trp mutant of the potassium channel KcsA (W67) using polarized time-resolved fluorescence measurements. For the first time, an analytical framework is reported to analyze the homo-Förster resonance energy transfer (homo-FRET) within a symmetric tetrameric protein with a square geometry. We found that in the closed state (pH 7), the W67-W67 intersubunit distances become shorter as the average ion occupancy of the SF increases according to cation type and concentration. The hypothesis that the inactivated SF at pH 4 is structurally similar to its collapsed state, detected at low K^+^, pH 7, was ruled out, emphasizing the critical role played by the S2 binding site in the inactivation process of KcsA. This homo-FRET approach provides complementary information to X-ray crystallography in which the protein conformational dynamics is usually compromised.

## Introduction

**Figure 1:**
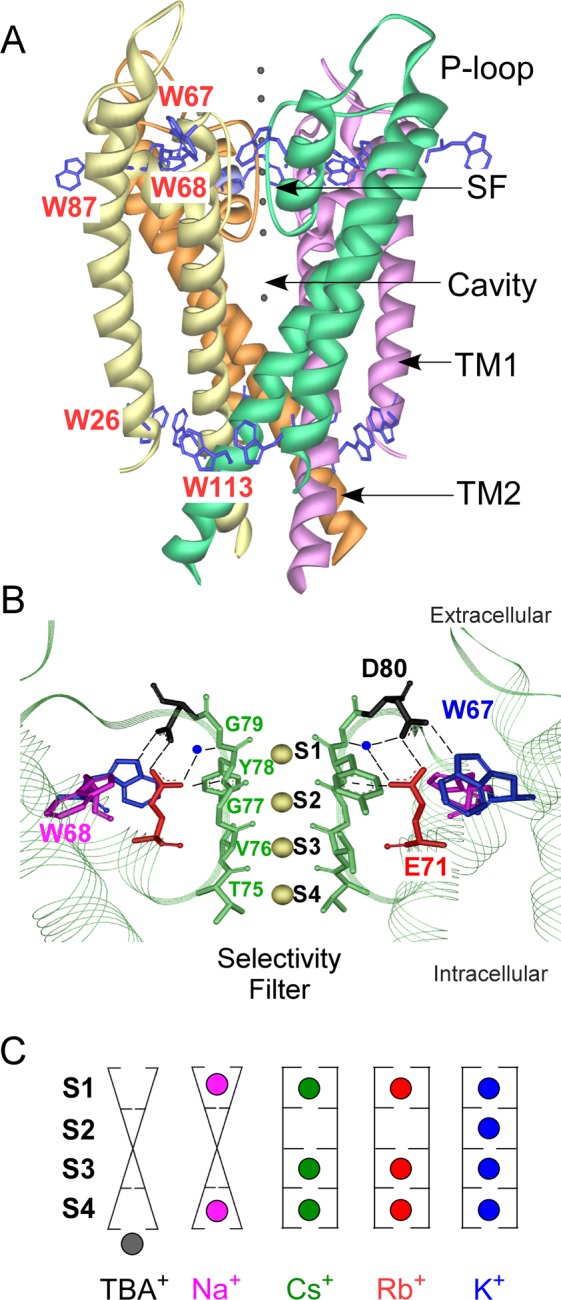
Structure of the wild-type KcsA channel. (**A**) A side-view of the tetramer is shown (PDB entry: 1K4C). Each monomer consists of two transmembrane helices (TM1 and TM2) connected by the P-loop region and the selectivity filter (SF). The five Trp residues per monomer are depicted as blue sticks. (**B**) Close-up of the selectivity filter of the KcsA channel. The W67 residue is located just behind the SF structure, participating in the inactivation triad, which involves a complex H-bonding network between D80, E71, W67 and extra interactions with Y78 and G79 from the SF. (**C**) Schematic representation of the SF binding sites that can be occupied by each ion at pH 7, according to X-ray crystallography available data^3,50^.

Potassium channels are integral membrane proteins present in prokaryotic and eukaryotic organisms where they contribute to the control of potassium flow, cell volume, release of hormones and neurotransmitters, resting potential, excitability, and behavior. Under physiological conditions these molecules are highly selective, allowing the permeation of K^+^ at near diffusion-limited rates, whereas Na^+^ is effectively excluded from passing through^1^. So far, the molecular basis of selectivity and permeation is still a matter of debate. KcsA is a proton-activated, voltage-modulated channel cloned from *S. lividans* and used as a prototypical protein on the biophysical studies of the K^+^ channels due to its simple structure: four identical subunits around a central pore, each one comprising an *N*-terminal domain, two transmembrane segments and the cytosolic *C*-terminal section. The two transmembrane segments (TM1 and TM2) are connected by a pore region that contains a tilted short-helix (pore helix), two loops and an ion selectivity filter (SF) (Fig. 1A). The SF contains four putative K^+^ binding sites delineated by the signature sequence TVGYG (Fig. 1B,C), clearly homologous to the more complex eukaryotic potassium channels and its conformational dynamics is mainly responsible for the permeation and the selectivity features of these membrane proteins^2,3^. Based on the conformation of the intracellular bundle (intracellular gate or gate I) and the SF (gate II), the gating cycle of KcsA is defined by at least four kinetic states: closed/conductive (pH7, high K^+^), open/conductive (transient state found in high K^+^ after the pH4 gating), open/inactivated (pH4, high K^+^, at steady state) and closed/inactivated (pH7, high K^+^, immediately after increasing the pH from 4 to 7)^4^. The opening movement at gate I is allosterically coupled to changes in SF conformation, inducing the slow inactivation process, and consequently leading to a steady-state, very low open probability of the wild-type (WT) channel^5,6^. The inactivation phenomenon after several milliseconds of activity occurs in a very similar way to eukaryotic channels^7,8^. Additionally, X-ray crystallography data from the closed state revealed two different conformations of the SF: a conductive conformation (Fig. 1B,C) in the presence of sufficient amounts of permeant cations (e.g. K^+^, Rb^+^ or Cs^+^), and a non-conductive or collapsed structure when in low concentration of permeant species or high quantities of blocking species such as Na^+^, with only the most external binding sites (S1 and S4) accessible to ion-protein interaction (Fig. 1C)^3,9,10^.

The SF conformation is also modulated by the amino acid residues that lie behind the signature sequence, especially those in the pore helix region, constituting a key scaffold component of the potassium channel^3,10^. The inactivation triad corresponds to the interaction between Glu71, Asp80 and Trp67 residues, forming a hydrogen-bond network that modulates the conductive/inactivated states by affecting the conformation of the K^+^-binding sites (Fig. 1B)^6^. In fact, mutation of Glu71 to alanine lead to a SF “frozen” in a conductive conformation in the presence of K^+^ and also Na^+^^11,12^. Macroscopic currents measured in this mutant channel revealed an almost abolished C-type inactivation process and consequently a high open probability. Some authors relate the non-conductive conformation of the SF seen in the X-ray experiments (PDB entry 1K4D) with the inactivated conformation^13^, since this phenomenon, is favored at low K^+^ concentrations in eukaryotic potassium channels^11,14–16^, whereas some other experimental information suggests only modest conformational changes at the G77 residue during inactivation^17^.

Here, we have used polarized steady-state and time-resolved fluorescence measurements to study tryptophan-tryptophan energy migration/Förster resonance energy transfer (FRET) in detergent-solubilized KcsA mutant channels to characterize the interplay between the pore helix and the SF conformation and its consequences over the different functional states of the protein. These measurements have the advantage of being performed at room temperature and using a low concentration of unlabeled protein, in contrast to X-ray crystallography, electron paramagnetic resonance (EPR) or nuclear magnetic resonance (NMR). Time-resolved fluorescence anisotropy is a powerful tool that has allowed to study homo-FRET in different systems and to determine distances and orientations between the fluorophores^18–22^. The extent of FRET depends on the reciprocal of the sixth power of the distance between donor and acceptor, making this technique a suitable “spectroscopic ruler” to measure distances within and between proteins^23^. When FRET occurs between identical fluorophores (homo-FRET or energy migration), the experimental observable is the fluorescence anisotropy of the sample because the successive reversible FRET steps lead to a strong depolarization of the fluorescence emitted by the molecule without any concomitant variation of its fluorescence intensity or lifetime^24,25^. In order to apply advanced FRET methodologies, a system with a high symmetry is required, so a KcsA mutant containing a single tryptophan residue per subunit is essential. The KcsA channel contains five tryptophan residues per subunit located at both ends of the transmembrane helical segments of the protein (Fig. 1A). W26 and W113 are located at the membrane-cytosol interface at the intracellular ends of the transmembrane segments; W87 is in contact with the lipid bilayer and W67 and W68 are buried in the channel core, all the latter at the extracellular side. The triple mutant channel W26,87,113F has already been shown to present ion conduction properties and spectroscopic characteristics very similar to the wild-type (WT) protein^26^. Moreover, the fluorescence signal from W67 and W68 residues are able to detect SF conformations (conductive or non-conductive) according to the type and concentration of cations^27^. These two residues are located at the pore helix, with their indole side chains practically in contact with the polypeptide backbone of the selectivity filter^2^. W67 is highly conserved among prokaryotic and eukaryotic potassium channels^28^ and, as mentioned above, is involved in C-type inactivation regulation^29^. Thus, this fluorescent residue was kept while W68 was mutated to phenylalanine in order to obtain a quadruple KcsA mutant with a single tryptophan residue per subunit (W26,68,87,113F or “W67” KcsA). We have also taken advantage of the non-inactivating E71A mutation to study the KcsA channel predominantly kept in a conductive conformation (non-inactivated state)^11^.

Our results indicate that W67 residues undergo an efficient homo-FRET process among the four channel subunits, and that their fluorescence anisotropy is an excellent reporter of the changes in the pore helix conformation induced by the progressive increase in the SF ion occupancy and by the gating transitions. Moreover, the analytical solution describing homo-FRET among a homo-tetramer in a square geometry allowed retrieving W67-W67 lateral distances from the W67 KcsA fluorescence anisotropy decays, shedding new light on the KcsA inactivated state conformation and its relationship to S2 SF binding site occupation.

## Results

### The W67 KcsA mutant behaves as a wild-type like protein and shows unique photophysical properties

**Figure 2:**
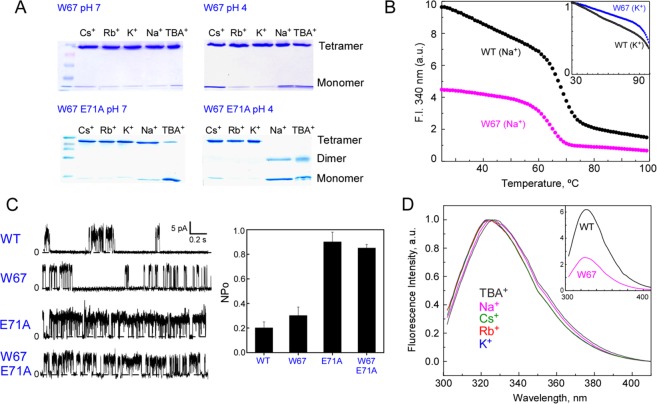
The structural and functional properties of the quadruple W67 KcsA mutant are similar to the ones presented by the wild-type channel. (**A**) SDS-PAGE (13.5%) analysis of the tetramer integrity of the detergent-solubilized protein in the presence of 20 mM Hepes, pH 7 buffer with 5 mM DDM and 200 mM Cs^+^, Rb^+^, K^+^ or 10 mM TBA^+^ or 10 mM succinic acid, pH 4 buffer with equivalent amounts of DDM and salts. In the particular case of Na^+^, 200 mM NaCl was added to the W67 KcsA samples and 1 M to the E71A W67 batches, respectively. pH 7 and pH 4 samples represent the closed and open states of the channel, respectively. (**B**) Protein thermal stability assay (monitoring changes in fluorescence emission at 340 nm) of 1 µM protein solubilized in 20 mM Hepes, pH 7 buffer with 5 mM DDM and 100 mM NaCl indicates that the introduced mutations induced only a 5 °C decrease in stability (t_m_: 66 °C) when compared to WT channel (t_m_: 70 °C). The inset reveals that in the presence of 100 mM K^+^, KcsA W67 is as highly resistant to thermal denaturation as the WT channel. (**C**) Patch-clamp inside-out patches recordings in continuous mode at + 150 mV of WT, W67, E71A and W67 E71A KcsA channels reconstituted in asolectin liposomes. The dashed lines indicate the closed channel state. Channel openings appear as upward deflections over the closed state line. Bar graphic illustrates open probability (NPo) determined at +150 mV. Data is presented as average ± SD of at least three independent experiments. (**D**) Normalized fluorescence emission spectra (λ_ex_ = 295 nm) of W67 KcsA mutant channel at pH 7 in the presence of different blocking and permeant cations. The inset compares the fluorescence emission spectra of WT and W67 KcsA mutant in the presence of 200 mM K^+^.

The presence of a predominant tetrameric structure of the detergent-solubilized W67 KcsA mutant channel was confirmed in the presence of 200 mM KCl, RbCl, CsCl and NaCl, at both pH 7 (closed state) and 4 (open state) (Fig. 2A). The tetrameric structure was also preserved in the presence of 5 mM tetrabutylammonium (TBA^+^) chloride. This blocker salt binds with nM affinity to the channel cavity, leaving the SF almost “empty” of cations^30^. The W26,68,87,113F substitutions had a minimal impact on the structural stability of the detergent-solubilized protein as shown by the thermal denaturation assays performed in the presence of either 100 mM Na^+^ or K^+^ at pH 7 (Fig. 2B). In addition, ion permeation properties tested by patch-clamp methods confirmed that W67 KcsA gates at pH 4 and behaved as the WT channel, showing a low open probability in the presence of K^+^ (Fig. 2C), thus validating this mutant channel as a wild-type like protein.

W67 KcsA remains a highly fluorescent protein, retaining 40–45% of the WT overall florescence intensity, due to the shielded position of its single tryptophan residue (W67) per subunit (protected from the aqueous solution) (inset to Fig. 2D). The fluorescence properties of W67 (blue-shifted emission spectra (maximum emission wavelength ca. 325 nm) and high quantum yield (Φ~ 0.3, using *N*-acetyl-*L*-tryptophanamide (NATA) in water as reference^31^) yielded a Förster radius of R_0_ = 12 Å, close to the highest values described in the literature for the tryptophan-tryptophan Förster pairs^20^. The steady-state (Fig. 2D, Table S1) and time-resolved (Table S2) spectral properties of W67 KcsA were slightly sensitive to the monovalent cation present in the buffer.

### Efficient inter-subunit homo-FRET occurs between the tryptophan residues of W67 KcsA channel depending on the ion occupancy of the SF

Direct inspection of several high resolution crystal structures of KcsA crystals in the absence of their C-terminal domains and with an antibody Fab fragment bound to the extracellular loop revealed W67-W67 lateral separation distances, *R* ~ 17–18 Å. In addition, *R* ~ 15.2–15.9 Å was found when working with the full-length channel bound to an intracellular Fab fragment (Table 1). These distances are close to the Trp-Trp Förster radius of *R*_0_ = 12 Å calculated from our spectroscopic data for the detergent-solubilized W67 KcsA and therefore intramolecular homo-FRET phenomena among the tryptophanyl residues within each channel is highly probable. To test this hypothesis, both steady-state and time-resolved fluorescence anisotropy measurements were carried out for the mutant channel under different ionic conditions and pH. The samples were excited at 300 nm as the fundamental anisotropy of tryptophan reaches its highest value (*r*_0_ ~ 0.3) at this wavelength^32^, allowing to maximize the dynamic range of the time-resolved measurements.

**Table 1:** Comparison between C(δ_2_)-C(ε_2_) inter-tryptophan lateral distances (*R*) obtained from X-ray crystallography and from analyzing the fluorescence anisotropy decays of the detergent-solubilized W67 and W67 E71A KcsA mutant channels with the simplified homo-FRET model (Eq. 1).

KcsA Channel	PDB entry	Fab	Presence of C-terminal domain	Ionic condition	X-ray data	Homo-FRET analysis	Δ*R* ^**^ (Å)
					*R*(Å)	R^*^(Å)	
“WT” Closed	—	n.a.	—	TBA^+^	—	19.5 ± 0.2	—
	2ITD	Extracellular	No	Ba^2+^	17.5	16.01 ± 0.01	1.5
	2ITC	Extracellular	No	Na^+^	18.1	17.6 ± 0.1	0.5
	1R3L	Extracellular	No	Cs^+^	17.5	16.45 ± 0.07	1.1
	1R3I	Extracellular	No	Rb^+^	17.3	15.88 ± 0.02	1.4
	1BL8	No	No	K^+^	17.3	15.4 ± 0.2	1.9
	1K4C	Extracellular	No	K^+^	17.3	15.4 ± 0.2	1.9
	3EFF	Intracellular	Yes	K^+^	15.9	15.4 ± 0.2	0.5
“WT” Open	3FB8	Extracellular	No	Rb^+^	17.6	16.7 ± 0.2	0.9
	3F5W	Extracellular	No	K^+^	17.7	15.3 ± 0.1	2.4
	3PJS	Intracellular	Yes	K^+^	15.2	15.3 ± 0.1	−0.1
E71A mutant	3OGC D80 flipped	Extracellular	No	Na^+^	23.9	14.9 ± 0.2	9.0
	—	n.a.	—	Cs^+^	—	15.32 ± 0.07	—
	—	n.a.	—	Rb^+^	—	15.4 ± 0.2	—
	1ZW1 D80 non-flipped	Extracellular	No	K^+^	17.5	15.23 ± 0.08	2.3
	2ATK D80 flipped	Extracellular	No	K^+^	24.5	15.23 ± 0.08	9.3

^*^The calculated distances represent the mean ± SD of at least three independent experiments performed at pH 7.

^**^Difference between the crystallographic W67 inter-tryptophan lateral distance and the average value recovered from the analysis of the time-resolved fluorescence anisotropy decays with the homo-FRET model derived assuming a square geometry.

n.a., not available.

**Figure 3:**
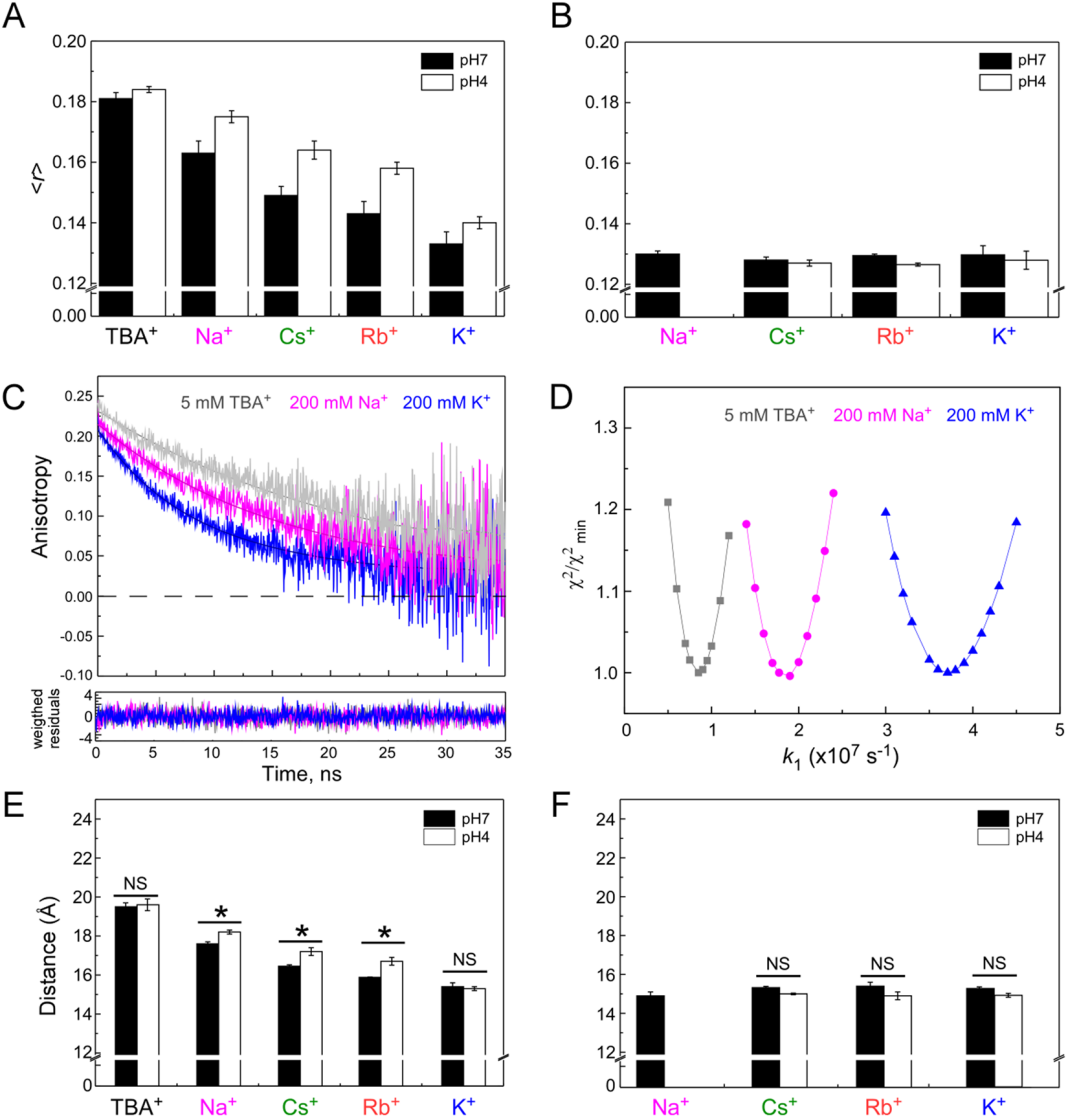
Conformational changes of W67 KcsA channel probed by steady-state and time-resolved anisotropy measurements. Steady-state fluorescence anisotropy values of 6 µM W67 (**A**) and W67 E71A (**B**) KcsA in 5 mM DDM solutions under saturating concentrations of monovalent cations, either 200 mM of Cs^+^, Rb^+^, K^+^ or 5 mM TBA^+^. The Na^+^ concentration used was 200 mM for KcsA W67 and 1 M for W67 E71A KcsA at pH 7. Measurements of both channels were carried out at pH 7 (closed state) or 4 (open state) **(C)** Representative fluorescence anisotropy decays, *r*(*t*), of W67 KcsA at pH 7 in the presence of 5 mM TBA^+^ (SF average ion occupancy: (0), 200 mM Na^+^ (SF average ion occupancy: (1) and 200 mM K^+^ (SF average ion occupancy: (2) (λ_ex_ = 300 nm; λ_em_ = 345 nm). The solid lines are the best fit of Eq. 1 to *r*(*t*) with _g_ = 43 ns kept as a fixed parameter in the analyses. The 95% confidence intervals calculated for *k*_1_ were [0.85,0.93]×10^7^ s^-1^, [1.87,1.94]×10^7^ s^-1^ and [3.60,3.83]×10^7^ s^-1^ in the presence of 5 mM TBA^+^, 200 mM Na^+^ or K^+^, respectively, at pH 7 (**D**) Dependence of the reduced χ^2^ on the energy transfer rate constant *k*_1_. The relative χ^2^ surfaces were obtained for the different ionic conditions by keeping *r*(0) as the only fitting parameter in Eq. 1. (**E,F**) represent the average lateral inter-W67 distances retrieved from fitting the anisotropy decays obtained for the detergent-solubilized W67 and W67 E71A KcsA channels, respectively, with the derived homo-FRET model. The data in panels A, B, E and F represent the mean ± SD of at least three independent experiments. Statistical significance was determined via two-way ANOVA with Bonferroni posttest ^*^P < 0.001 for pH 7 relative to pH 4. *P* values greater than 0.05 were considered not significant (NS).

At pH 7, the steady-state anisotropy of W67 KcsA showed a strong dependence with the cation present in the bathing solution: the more permeant the monovalent cation is, the lower the steady-state anisotropy presented by the protein (<*r*>_K_^+^ ~ 0.135 ± 0.004 (*n* = 5) <<<*r*>_Rb_^+^ <<*r*>_Cs_^+^ <<<*r*>_Na_^+^ ~ 0.166 ± 0.004 (*n* = 5)). The highest steady-state anisotropy was obtained in the presence of 5 mM TBA^+^, *i.e*. with an “empty” SF ($< r{ > }_{{{\rm{TBA}}}^{+}}$ ~ 0.180 ± 0.002 (*n* = 3)) (Fig. 3A). Altogether, these data strongly suggest that the more permeant cations induce local conformational changes in the channel at the SF and pore helix level, hence increasing the efficiency homo-FRET among the tryptophanyl residues and consequently producing a stronger depolarization of the protein fluorescence (lower <*r*> values). This hypothesis was corroborated by the time-resolved anisotropy data obtained for the mutant channel. These measurements are fundamental to retrieve quantitative structural information (distances and orientations) from systems undergoing homo-FRET^18–21^. The anisotropy decay of W67 KcsA became much faster in the presence of K^+^, the more permeant cation, as compared to the blocking agents TBA^+^ and Na^+^, a result that is a spectroscopic signature for the occurrence of an efficient homo-FRET process (Fig. 3C)^25^.

**Figure 4:**
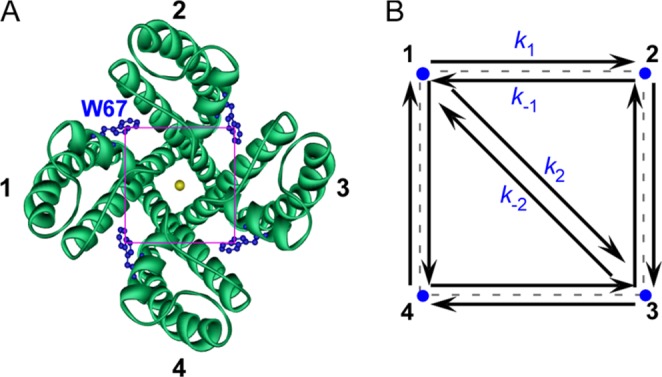
Topological model used in the determination of lateral inter-W67 distances by homo-FRET measurements. (**A**) Top view of the KcsA channel (PDB entry: 1K4C) with each W67 residue depicted as blue sticks. The subunits of the tetrameric channel were arbitrarily numbered 1, 2, 3 and 4. (**B**) Diagram for homo-FRET within a square geometry. The W67 residues in the native symmetric homotetrameric channel define a square with lateral distance, *R*, and a diagonal distance between opposite monomers across the central pore, ${R}_{2}=\sqrt{2\,}R$. Considering that the initially excited W67 residue is at subunit 1, *k*_*i*_ and *k*_*−i*_ are the Förster′s transfer rates for this residue.

The four-fold axis of symmetry of the KcsA channel and consequently the square geometry delimited by the W67 residues (Fig. 4), allowed deriving a formalism describing the fluorescence anisotropy decay of the W67 KcsA mutant-DDM complex. As detailed in the Theory section (SI), the depolarization of the fluorescence emitted by the W67 residues was considered to be due to two main processes: (i) the overall rotational tumbling of the tetrameric KcsA-DDM complex in buffer solution, and (ii) energy transfer between the subunits within each tetrameric channel. This model has three fitting parameters, namely the initial anisotropy of each W67 residue, *r*(0), the FRET rate constant between neighbouring Trp residues, *k*_1_, (Fig. 4), and the global rotational correlation time, *ϕ*_g_, of the KcsA-DDM complex (Eq. 1). This rotational correlation time was first determined independently by measuring the time-resolved anisotropy decay of KcsA L90C labeled with a long-lived fluorescent probe, *N*-1-pyrene-maleimide (see SI for a detailed description of the process); a *ϕ*_g_ = 43 ± 3 ns was obtained and fixed during the subsequent fitting procedures. Then, we investigated whether changes in ionic conditions (use of different blocking/permeant cations at saturating concentrations) influenced the tryptophan-tryptophan lateral distance *R* within each channel. The derived homo-FRET formalism (Eq. 1) was fitted to the fluorescence anisotropy decays obtained for W67 KcsA at pH 7 (closed state at the inner gate). As exemplified in Fig. 3D, the relative^2^ surfaces showed well-defined minima and therefore the fittings were adequate to determine the lateral rate constant *k*_1_. Using Eq. 2, the intramolecular lateral inter-tryptophan distances, *R*, could now be retrieved from the fitted rate constants *k*_1_. As tryptophan is an intrinsic fluorescent residue of the proteins, the distances calculated by the homo-FRET approach can be directly compared to those obtained from the WT KcsA crystal structures. The results indicate that at pH 7.0, the calculated distances gradually decreased (*R*_Na+_ > *R*_Cs+_ > *R*_Rb+_ > *R*_K+_ (Fig. 3E and Table 1)) upon increasing the ion occupancy of the SF, and therefore with the permeability sequence already described for the WT channel^27^. Remarkably, the larger and the shorter average W67-W67 Cδ2-Cε2 lateral distance within the protein were estimated to be ~19 Å in the presence of 5 mM TBA^+^, when the SF is essentially empty^23^, and ~15 Å in the presence of 200 mM K^+^, when the average ion occupancy of the SF is ~2, respectively, illustrating the conformational plasticity of this domain when the potassium channel is in solution at room temperature (Δ*R*_max_ ~ 4 Å (Table 1)). In contrast, W67-W67 Cδ2-Cε2 distances calculated from the X-ray data available from the truncated channel (Δ125) (bound to an Fab segment tagging the extracellular loop) show only small differences in the presence of different conducting and blocking cations (*R*~ 17–18 Å and therefore Δ*R*_max_ < 1 Å (Table 1)). Interestingly, the homo-FRET derived W67-W67 lateral distance in the presence of K^+^ was found to be almost identical to the distance retrieved from the X-ray data obtained only for the full length channel bound to a stabilizing “C-terminal domain” antibody (PDB entry: 3EFF, Table 1). This is clear evidence that the conditions required for X-ray studies critically affect the structure of the channel, imposing restrictions to the conformational changes at the SF level.

**Figure 5:**
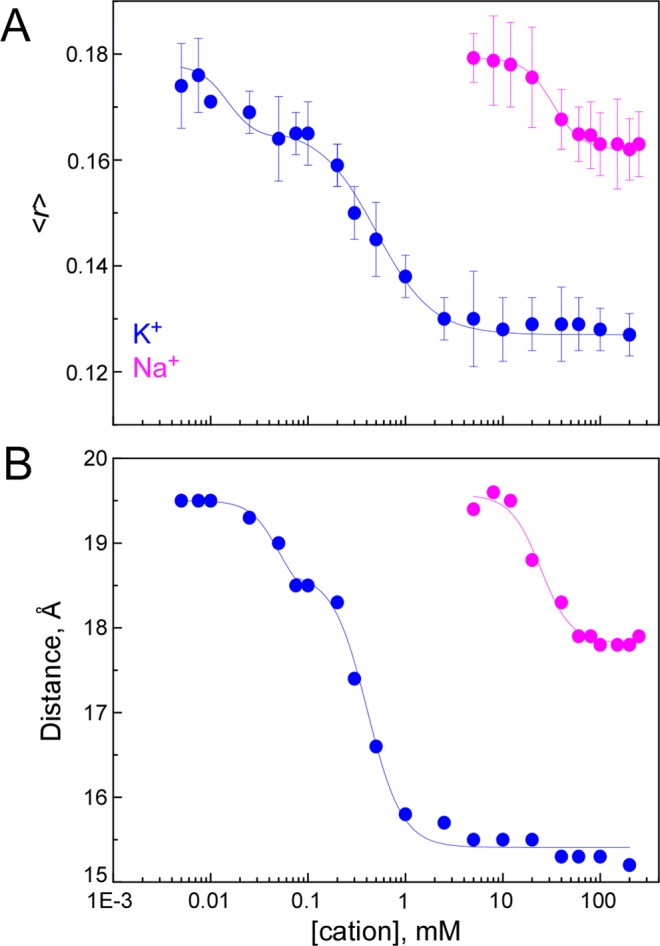
Conformational dynamics of the detergent-solubilized W67 KcsA channel as a function of the ion occupancy of its selectivity filter. Dependence of the (**A**) steady-state fluorescence anisotropy, <*r*>, and (**B**) lateral inter-W67 distances of the mutant channel on K^+^ and Na^+^ concentration. The distances were retrieved from fitting the anisotropy decays obtained for 6 μM W67 KcsA mutant channel with the derived homo-FRET model (Eq. 16) (λ_ex_ = 300 nm; λ_em_ = 345 nm;). The conditions used were pH 7 buffer (closed state) containing 5 mM DDM and varying concentrations of K^+^ (blue) or Na^+^ (magenta). As already described for the WT channel^26,33^, two consecutive binding events with different affinities could also be detected upon K^+^ binding to W67 KcsA mutant channel. For Na^+^, only a subtle stabilization of the tetrameric mutant channel could be observed upon increasing its concentration in solution. The solid lines are just a guide to the eye. The concentration range accessible experimentally for this cation was much narrower due to the dissociation of the DDM-solubilized W67 KcsA channel below 1-2 mM of Na^+^.

To further test the conformational dynamics of the short pore helices, we took advantage of the well-known conformational equilibria of the SF, which is able to interconvert between a collapsed, non-conducting conformation, and a conductive structure, where all the four binding sites for K^+^ are detected. At pH 7 this equilibrium is regulated by the type and concentration of the cation present in the filter, since only sufficient amounts of permeant cations are capable to shift the equilibrium towards the SF conductive state. Moreover, these conformations have been described to have different affinities for K^+^ or Na^+^^26,27^. Here, we were able to monitor the transition between these conformational states by performing both steady-state and time-resolved anisotropy measurements of W67 KcsA as a function of either K^+^ or Na^+^ concentration, at pH 7. Both the steady-state fluorescence anisotropy (Fig. 5A) and the calculated inter-subunit W67-W67 lateral distances obtained from analyzing the corresponding time-resolved data with the derived homo-FRET formalism (Fig. 5B) varied pronouncedly in a dose-response manner with either the K^+^ or Na^+^ concentration used. These results reveal how powerful the homo-FRET approach here described is, since it is able to verify previous thermodynamic studies performed with the WT KcsA channel solubilized in DDM, where K^+^ binding to the protein has been described by two consecutive binding events, the first one with an apparent 1000-fold higher affinity than the second one^26^. The first binding event, which corresponds to the stabilization of the non-conducting structure, in which K^+^ is able to bind either to S1 or S4 SF binding sites (with an average occupancy of just one K^+^ distributed between these two sites)^3^, resulted here in a ~1 Å decrease in the calculated average inter-tryptophan lateral distances (Fig. 5B). Further increasing the concentration of the permeant K^+^ cation leads to a higher occupancy of the pore, as a second ion goes into the middle of the filter (site S2 or S3), self-inducing the formation of the conductive structure. This conformation shows a lower affinity for the cation in order to allow for the high-efficiency K^+^ permeation process to occur^26^. As it is shown in Fig. 5B, this second step further narrowed down the average inter-tryptophan neighboring distances between the four subunits of W67 KcsA by ~3 Å. A similar titration experiment was also conducted with Na^+^, where the concentration-dependent response of both <*r*> and *R* was now monophasic, resulting in an overall Δ*R* ~ 1.5–2 Å, also in agreement with the previously described binding of Na^+^ to the most external site (S1)^33^.

### Ion occupancy at S2 binding site controls the modest conformational changes linked to pH-induced gating of W67 KcsA

We next sought to gain some insight into the conformational changes at the SF and pore helix derived from the pH-induced gating of the channel and its relationship to the C-type inactivation process that takes place in the presence of K^+^ once the intracellular gate is open. With this objective in mind, fluorescence anisotropy measurements made for the W67 KcsA mutant channel at pH 7 (closed state) and 4 (open state) were compared. Figure 3 (panels A and E) show that both the steady-state anisotropy and average lateral inter-tryptophan distances presented the same general trend at both pH values as the two parameters gradually decreased upon replacing the blocking agents TBA^+^ or Na^+^ with the progressively more permeant cations Cs^+^, Rb^+^ and K^+^, respectively. More importantly, triggering the opening of the inner bundle gate generated only a modest change on the anisotropy decays (Fig. S1), and consequently on the average inter-subunit W67-W67 lateral distances between the open and closed states (Δ*R* ~ 0.6–0.8 Å). The difference between the two distances was found to be statistically significant (*P* < 0.001 (pH 7 vs pH 4) as determined from two-way ANOVA (with Bonferroni multiple comparison posttest), only in the presence of saturating concentrations of Na^+^, Cs^+^ and Rb^+^ (Fig. 3E). Analysis of full length KcsA X-ray data also revealed that in the presence of high amounts of K^+^, there is no significant variation in W67-W67 lateral distances when comparing the closed and open states at the inner gate (Table 1). It should be stressed that there is no structural information available for any other cation with this experimental approach.

At saturating cation concentration, the main difference between the KcsA complexes with Na^+^, Cs^+^, Rb^+^ versus K^+^, is the ability of the latter to occupy the S2 position at the SF^10^, so we decided to test the hypothesis that S2 binding site occupancy would lead to shorter W67-W67 lateral distances and insensitivity to acid pH gating. To this end, barium binding to the mutant channel was also evaluated. Ba^2+^ is a divalent cation which acts as universal blocker of K^+^ channels by binding to the selectivity filter with high affinity (nM range). KcsA X-ray crystallographic data (PDB entry: 2ITD) revealed that WT KcsA can complex barium cations not only at S4 but also at S2 binding site, leaving both positions S1 and S3 empty. As shown in Fig. S1, W67 KcsA presented similar steady-state and time-resolved fluorescence anisotropies in the presence of saturating concentrations of Ba^2+^, at both pH values. The change in the W67 inter-subunit distance between the open/inactivated and closed states of the mutant channel was now non-significant (*P* > 0.05 (pH 7 vs pH 4) as determined from two-way ANOVA with Bonferroni multiple comparison posttest), suggesting that occupation of the S2 SF binding site precludes a pH-induced conformational change at the W67-W67 lateral distance level.

### E71A mutation locks W67 E71A KcsA in a conductive conformation

The second approach used here to explore the structural changes linked to channel inactivation was to study the behavior of W67 E71A KcsA. The E71A mutation exhibits a non-inactivating phenotype since it abrogates the C-type inactivation process, presumably because its SF is stabilized in a conductive conformation, even in the presence of low K^+^ concentrations or high Na^+”^^11,12^. The E71A W67 KcsA mutant behaved in a similar way to the E71A counterpart (see Fig. S2), so we expected the anisotropy decays of the W67 E71A mutant channel to resemble the one obtained for the conductive conformation of W67 KcsA in 200 mM KCl buffer. As exemplified in Fig. S2, this prediction was experimentally confirmed as the inter-subunit W67 distances calculated for W67 E71A KcsA were ~15 Å under all conditions tested (in the presence of 200 mM K^+^, Rb^+^, Cs^+^ and 1 M Na^+^ at both pH (Fig. 3F)), close to the values previously obtained for W67 KcsA in 200 mM KCl, when homo-FRET among the tryptophan residues was most efficient (Fig. 3E).

Crystal structures of the closed state of the E71A channel at high K^+^ concentration show that D80 and W67 residues could adopt two different conformations: one that resembles the WT structure (“non-flipped”) and a “flipped” structure where there is disruption of the H-bond between these two residues leading to a rotation of the W67 side chain “away” from the SF (Fig. S3A)^11^. It should be noticed that all distances here determined were not compatible with the presence of the “flipped” conformer of the potassium channel. The data obtained here are also in agreement with previous thermal stabilization studies carried out with E71A KcsA^34^.

## Discussion

### The conformation of the short pore helices is strongly correlated with SF ion occupancy of W67 KcsA in the closed state

The location of W67 in the short pore helix allowed studying the interplay between the SF occupancy and pore helix conformation. The W67-W67 inter-subunit lateral distances were found to be sensitive to cation-type and cation concentration within the pore. At saturating salt concentration, the larger W67-W67 distances were found when the blocker TBA^+^ was used, as expected for a cation that only binds to the channel cavity, barely interacting with an “empty” SF at the T75 level. As the SF ion occupancy increases (Na^+^ < Cs^+^~Rb^+^ < K^+^), the tryptophan residues present in each subunit of the W67 KcsA channel become closer to each other, the inter-subunit FRET rate constants increased and the excitation energy was more effectively distributed among the four tryptophan residues of the channel which, in turn, led to a stronger depolarization of the emitted fluorescence. The KcsA X-ray data (PDB entry 1K4C and 2ITC) show a tendency of the protein subunits to get closer to each other at the SF level in the presence of high K^+^ compared to high Na^+^ (even though to a lesser extent than the channel in solution), and previous EPR experiments also revealed that channel subunits gather around the central pore during the C-type inactivation process^35^. Thermal denaturation assays performed in the detergent-solubilized WT channel also describe that cations can bridge together the protein subunits by interacting through multiple contacts with the internal cavity and the SF^26^. Based on these evidences, we claim that changes in W67-W67 lateral distances seen in these homo-FRET experiments are mainly due to the movement of the pore helices approaching each other towards the pore axis, even though modifications on Trp side chain angle (away/closer to the SF, in a similar way to the flipped conformation of E71A mutant) cannot not be dismissed.

Regarding the physiologically relevant K^+^ ion, our data provide additional experimental evidence for the nonconductive-to-conductive conformational change undergone by the KcsA channel upon increasing the total number of ions in the filter, as previously described in the Introduction section. This equilibrium between the two dominant channel structures is driven by the association of the second ion to the central binding sites (sites S2 or S3) in the singly occupied filter. The homo-FRET dose-response experiments detected two consecutive K^+^ binding events, the first one bridging closer together the channel subunits by ~1 Å and the second one by ~3 Å respectively. As previously seen in the WT channel^26^, these two consecutive transitions correspond to the stabilization of the non-conductive state and to the induction of the conductive conformation of the SF, respectively. The greatest change in the intersubunit lateral distances observed during the second binding event of K^+^ correlates well with the vast thermal stabilization observed during this process (35–40 °C) in the WT channel^26^. These experiments also reveal that the steady-state fluorescence anisotropy of our systems, a much more accessible experimental parameter than the determination of the rate constant *k*_1_ via time-resolved anisotropy measurements, can be used directly as a spectroscopic ruler of the average conformation adopted by W67 KcsA in solution.

**Figure 6:**
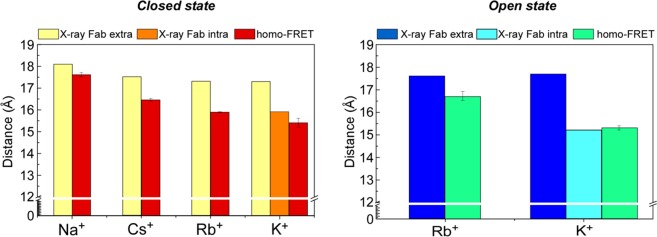
Comparison between the lateral inter-W67 distances calculated from fitting the anisotropy decays obtained for the detergent-solubilized W67 KcsA channels with the derived homo-FRET model (Eq. 1) and the same distances obtained from the X-ray data available for the closed and open states of KcsA channel. High resolution crystallographic structures include those obtained in the presence of the Fab segment against the extracellular loop (Fab extra) or the C-terminal domain (Fab intra), as presented in detail in Table 1. Homo-FRET measurements were carried out at pH 7 (closed state) or 4 (open state) in 5 mM DDM solutions under saturating concentrations of monovalent cations, either 200 mM of Cs^+^, Rb^+^, K^+^ or Na^+^. The data represent the mean ± SD of at least three independent experiments.

Finally, it is important to highlight that the overall Δ*R* that was obtained from the homo-FRET measurements upon varying the cation type/concentration in solution was much larger than the one calculated from the available crystallographic data for the truncated channel stabilized externally with a bound Fab antibody fragment (Fig. 6 and Table 1). Several factors may contribute to these discrepancies: first, our measurements were performed with a very low concentration of the detergent-solubilized, full-length mutant W67 KcsA at room temperature, when the channel is expected to display a much larger conformational freedom than in its crystal form. In fact, crystal packing forces near the SF might contribute to the lack of significant structural alterations in this channel region. Secondly, the auxiliary antibody Fab fragments included in the crystallographic studies were absent from our experiments. Depending on different authors, the Fab fragment is either directed towards the channel extracellular domain, involving interaction with the pore helix R64 and nearby residues^11^, or towards the intracellular C-terminal^36^. Both co-adjuvants have already been shown to reduce the rate of channel entry into the inactivated state coupled to pH-induced gating (more noticeable in the “extracellular” Fab segment). The homo-FRET derived distances obtained in the presence of saturating concentrations of K^+^ are indeed more similar to the ones reported for the full length KcsA crystallographic data (PDB entry: 3EFF (Table 1)), further hinting that the Fab fragment bound to the channel ectodomain is restraining its conformational freedom. In addition, comparison of the X-ray data from the full length channel attached to a Fab segment (PDB entry: 3EFF, *R* = 15.9 Å, Table 1) and the truncated KcsA channel without any antibody (PDB entry: 1BL8, *R* = 17.3 Å, Table 1) also hints to a possible allosteric effect induced by chymotrypsin cleavage of the C-terminal domain as an additional factor responsible for the discrepancies detected between the homo-FRET and X-ray calculated distances. Hence, the high-resolution structures provided by protein crystallography may not always reflect the predominant structure displayed by the protein in less extreme experimental conditions (i.e., at low concentration in aqueous solution at room temperature).

### The “conductive” E71A mutant freezes the W67 position in the “non-flipped” conformation

As previously described, the substitution of a glutamate residue by the smaller alanine residue at position 71 completely abolished the C-type inactivation process, leaving the SF “frozen” in a conductive conformation, not only in the presence of K^+^ but also Na^+^^11,12^. According to molecular dynamics simulations, the interaction between E71 and D80 residues contributes to the stabilization of the pore helix and the SF, and the strength of this interaction could be associated to movements of this helix. On the other hand, the H bonding between D80 and W67 would be less stabilizing in terms of channel structure, but more relevant since is more conserved among the K^+^ channel family^37^. The lack of SF flexibility in this mutant was further corroborated by our analysis, where the calculated inter-subunit lateral distances were now essentially the same whether Na^+^, K^+^, Rb^+^ or Cs^+^ ions were present at both pH7 and 4.0. Previous thermal denaturation experiments performed on this mutant also described a very similar behavior in the presence of these permeant cations^38^, and radioactive flux assays also demonstrated a decrease in cation selectivity induced by the E71A mutation in KcsA^12^. Apparently, upon disruption of the hydrogen bond between E71 and D80 from the inactivation triad, the SF of this mutant channel maintains a permanently conductive conformation, which is able to accommodate monovalent ions with very different sizes while displaying very similar lateral W67-W67 distances at the level of the short pore helices of the mutant channel. Furthermore, all the calculated distances now resemble those obtained from the W67 channel in the presence of K^+^, confirming the similarity between all the “conductive” states in terms of SF and pore-helix conformation. Cuello *et al*. recently explored the open conformation of a truncated form of the E71A mutant^39^. The reported X-ray crystallography data in the presence of K^+^ showed a very similar conformation of the SF compared to the resting state, supporting our homo-FRET results at pH 4, and pointing out to a modification in the strength of the interaction between the SF carbonyl groups and the K^+^ cations induced by channel gating in this mutant.

As previously mentioned, X-ray crystallography experiments also revealed two possible W67 conformers in the closed E71A mutant: a “non-flipped” conformation, very similar to that seen in the WT channel, and a “flipped” position of the D80 residue, oriented extracellularly, and leaving the W67 free to move away from the channel core. However, the distances calculated from our experiments support only the presence of the “non-flipped” channel structure, since a strong reduction in the homo-FRET process would be expected for the “flipped” conformer (i.e., a larger W67-W67 lateral distance should be found). Cordero-Morales and collaborators found that the stabilization of D80 in the flipped conformation, and consequently the loss of the interaction with W67, is probably due to an electrostatic interaction with Arg93 in the Fab segment, helping to stabilize that particular conformation of the filter^11^. Taking all these data into account, our results indicate that the W67-D80 H bond interaction is stable at room temperature in the detergent solubilized system in the absence of any antibody segment, and the mutation of E71 residue to alanine is not sufficient to disrupt this bond under the described conditions.

### The X-ray collapsed state does not account for the conformation of the inactivated state

As a proton activated channel, buffer exchange to decrease the pH from 7.0 to 4.0 triggers the opening of the KcsA channel at the intracellular gate and its subsequent inactivation. During the last years, attempts have been made to characterize the inactivated state of KcsA. Whereas some authors propose that the “collapsed” state, initially seen by X-ray crystallography at low K^+^ concentration (PDB entry: 1K4D), accounts for the conformation of the SF in the inactivated state^39–41^, new pieces of information indicate that structural modifications linked to the inactivated state are not equivalent to that described for the collapsed state at low K^+^, including different binding affinities for this cation and modifications on the ion occupancy at the most extracellular S1 binding site^17,42–44^ Comparison between the distances determined in this work for the closed/conductive (pH 7, 200 mM K^+^, *R* = 15.4 Å), closed/non-conductive (pH7, 0.1 mM K^+^, *R* = 16.5 Å) and open/inactivated (pH4, 200 mM K^+^, *R* = 15.3 Å) states clearly rule out the hypothesis that the inactivated state is structurally similar to its collapsed state, in agreement with recent studies.

It should also be stressed that once again, W67-W67 lateral distances recovered from the X-ray crystallography data show significant differences when comparing the open channel in the presence of a Fab segment bound to the extracellular face (PDB entry: 3F5W, *R* = 17.7 Å (Table 1)) or to the intracellular C-terminal domain (PDB entry: 3PJS, *R* = 15.2 Å (Table 1)). The distances for the W67 mutant channel calculated from the anisotropy decays in the presence of K^+^ at pH4 are in accordance with those observed in the crystals of the full length channel bound to the intracellular antibody. Moreover, comparison between the W67 inter-subunit crystallographic distances from the full-length KcsA channel in the closed and open states (PDB entries 3EFF and 3PJS, respectively) shows no significant changes, indicating that there is a fine and subtle tuning of KcsA conformation which modulates the inactivation of K^+^ channels.

Finally, a more detailed analysis of our results reveals that the relative changes in the homo-FRET distances (Δ*R* pH7 versus pH4) depends on the type of cation present in the solution: whereas Na^+^, Rb^+^ and Cs^+^ showed a statistically significant small increase in *R*, no significant modification was found in the presence of K^+^ or Ba^2+^. As previously described throughout the manuscript, K^+^ and Ba^+^ share the ability to bind to the S2 binding site of the SF (among others). This maintenance in the recovered distances using the W67 from the pore helix as a reporter suggests that the occupation of the S2 site lead to a very similar conformation of the SF in the open and closed states. Additionally, the ion occupancy at S2 also seems to be relevant in terms of the C-type inactivation process: Cs^+^ and Rb^+^ are permeant species that cannot bind to S2 site in the SF, a result that has been correlated to a 1000-time slower C-type inactivation process and 20-fold increase in mean open times (compared to K^+^) in the WT channel^17,38^. Here, Rb^+^ and Cs^+^ are the only permeant species that showed a small but significant increase in the W67-W67 lateral distances when the channel is in the open state. Interestingly, the increase in the W67 inter-subunit distances from pH 7 to pH 4 suggests that the open-conductive (pH 4) structure of the channel is not exactly equal to the closed-conductive (pH 7) conformation when working with these two cations. These results advocate that in the presence of Rb^+^ and Cs^+^ the interaction of the inactivation triad is somehow “less stable” and gives the W67 more conformational freedom, whereas the binding of K^+^ to two out of the four putative binding sites in the SF stabilizes the inactivation triad both in the closed (pH 7) and open (pH4) states. Matulef *et al*.^42,45^ also suggested that occupation of S2 SF binding site is a key element for the C-type inactivation process to occur in K^+^ channels.

In sum, the homo-FRET analysis has provided a quantitative picture of the changes in inter-subunit distances (lateral inter-tryptophan distances) at the level of the pore helices of the W67 KcsA mutant channel upon ion binding and pH gating, with an uncertainty of about +/−0.2 Å. Furthermore, comparison with X-ray crystallographic distances highlights how the presence of a bound Fab fragment and/or the hydrolysis of the C-terminal domain modify the extracellular loop and the SF conformation. In the short term, we plan to extend the homo-FRET studies to evaluate how the lipid environment modulates the structural plasticity of KcsA, a topic that it is difficult to explore by the X-ray crystallography approach.

## Methods

For a detailed description of materials and methods used, please see S.I. section.

### Protein expression, purification and samples preparation

Briefly, KcsA W26,68,87,113F (W67); W26,68,87,113F E71A (W67 E71A) and L90C mutant channels were purified in *E.coli* M15 (pRep4) heterologous expression system and purified by Ni^2+^/His-tag affinity chromatography according to previous reports^46^. Purified channels were then dialyzed against the pH 7 (20 mM HEPES, 5 mM n-Dodecyl-β-D-maltoside (DDM), 5 mM *N*-methyl-*D*-glucamine) or pH 4 (10 mM succinic acid, 5 mM DDM, 5 mM *N*-methyl-*D*-glucamine) buffers supplemented with the adequate concentration of KCl, RbCl, CsCl, NaCl, BaCl_2_ or tetrabutylammonium chloride (TBA.Cl) salts. Unless otherwise indicated, the typical protein concentration used in spectroscopic assays was 6 µM (monomer based). For patch-clamp functional exploration, KcsA was reconstituted into asolectin liposomes at 100:1 lipid to protein ratio (w/w) using Bio-Beads SM-2^47^.

### Fluorescence measurements and analysis

Intrinsic fluorescence emission spectra and steady-state fluorescence anisotropy were measured on a Horiba Jobin Yvon Fluorolog-3-21 spectrofluorometer. Time-resolved fluorescence and anisotropy measurements with picosecond resolution were obtained using the time-correlated single-photon timing (SPT) technique. The fluorescence decays of the 6 μM detergent-solubilized W67 KcsA samples (λ_exc_ = 300 nm) and pyrene-labeled L90C KcsA channels (λ_exc_ = 335 nm) were measured at 345 nm and 400 nm, respectively, using an emission polarizer set at the magic angle (54.7°) relative to the vertically polarized excitation beam produced by a frequency doubled Rhodamine 6 G laser, as previously described^48^.

Analysis of the fluorescence intensity decays were performed with the TRFA software (Scientific Software Technologies Center, Minsk, Belarus). The anisotropy decays of the detergent-solubilized W67 and W67 E71A KcsA samples were analyzed as (see S.I. for details):

1 $$r(t)=\frac{r(0)}{4}[1+\exp (-4{k}_{1}t)+2\exp (-\frac{9}{4}{k}_{1}t)]\cdot \exp (-t/{\varphi }_{g})$$

where *r* (0) is the initial anisotropy, *k*_1_ is the rate constant for FRET (between neighboring tryptophan residues (Fig. 3B) and *ϕ*_*g*_ is the rotational correlation time of the KcsA-DDM complex (independently determined using pyrene-labeled L90C KcsA). The inter-tryptophan lateral distance *R* can be directly calculated via *k*_1_

2 $${k}_{1}=\frac{1}{\tau }{(\frac{{R}_{0}}{R})}^{6}$$

where τ is the intensity-weighted mean fluorescence lifetime, and *R*_0_ is the critical radius computed with an orientational factor *κ*^2^ = 2/3.

The time-resolved anisotropy measurements of the pyrene-labeled L90C KcsA channels solubilized in the detergent micelles were globally analyzed using a two-step procedure using the TRFA software as previously described^49^. The usual statistical criteria, namely a reduced χ^2^ < 1.2 and a random distribution of weighted residuals and autocorrelation plots, were used to evaluate the goodness of the fits^31^.

### Statistics

Statistical analysis was performed using Graphpad Prism version 5 (Graphpad Software). The statistical significance of observed differences was assessed by one-way or two-way ANOVA with posttest as indicated. Results are expressed as mean ± standard deviation, S.D.

## Supplementary information

Supplementary information
