# Supplementary material for: Conformational plasticity in the KcsA potassium channel pore helix revealed by homo-FRET studies
Source: Sci Rep. 2019 Apr 17;9:6215. doi: 10.1038/s41598-019-42405-5 (PMC6470172; doi:10.1038/s41598-019-42405-5)
Supplement: Supplementary file 1 — Supplementary information [file 41598_2019_42405_MOESM1_ESM.docx]

**Conformational plasticity in the KcsA potassium channel pore helix revealed by homo-FRET studies**

M. Lourdes Renart, A. Marcela Giudici, José A. Poveda, Aleksander Fedorov, Mário N. Berberan-Santos, Manuel Prieto, Clara Díaz-García, José M. Gonzalez-Ros, Ana Coutinho.

**Supplementary information**

**EXPERIMENTAL PROCEDURES**

**Materials**

*N*-Dodecyl-β-*D*-maltoside (DDM) ULTROL® Grade was from Calbiochem (Life Technologies). Hepes acid, succinic acid, KCl, RbCl, CsCl, NaCl, BaCl_2_, tetrabutylammonium chloride (TBA.Cl), *N*-acetyl-*L*-tryptophanamide (NATA), *N*-mehtyl-*D*-glucamine (NMG), dimethyl sulfoxide (DMSO) and Tris(2-carboxyethyl)phosphine hydrochloride (TCEP) were from Sigma-Aldrich. Ni^2+^-Sepharose Fast Flow resin was from GE Healthcare, Bio-Beads SM-2 from Bio-Rad laboratories and *N*-(1-pyrene) maleimide was purchased from Molecular Probes (Life Technologies). Asolectin (*L*-α-Phosphatidylcholine from soy) was obtained from Avanti Polar Lipids.

**Protein mutagenesis, expression, purification and reconstitution**

W26,68,87,113F; E71A and L90C mutations were introduced on kcsA gene by PCR site-directed mutagenesis using KOD Hot Start DNA Polymerase (Merck) in order to obtain the final mutant proteins KcsA W26F,W68F,W87F,W116F (quadruple mutant, so-called W67 KcsA); W26F,W68F,W87F,W116F,E71A (quintuple mutant, named as W67 E71A KcsA) and L90C KcsA (single mutant). Mutations were confirmed by dideoxynucleotide sequencing.

Mutant channels heterologous protein expression was carried out in *E.coli* M15 (pRep4) and purification by Ni^2+^/His-tag affinity chromatography was performed according to previous reports.^1^ Protein concentration (always expressed in terms of monomer along the article) was routinely determined from the absorbance at 280 nm using a molar extinction coefficient of 12,950 M^-1^cm^-1^ and 34,950 M^-1^cm^-1^ for the W67 and W67 E71A KcsA monomers and for the L90C mutant, respectively, as estimated from the extinction coefficients of model compounds. ^2^

The final step after protein purification consisted in a dialysis against 20 mM HEPES, pH 7 buffer (pH 7 buffer/ closed state) or 10 mM succinic acid, pH 4 buffer (pH 4 buffer/ open state), each containing 5 mM DDM and the desired concentration of the following salts: TBA.Cl, BaCl_2_, KCl, RbCl, CsCl or NaCl. The final pH of each buffer was adjusted by adding *N*-methyl-*D*-glucamine. For the functional assays, the protein aliquots were reconstituted in lipid vesicles composed of asolectin as previously described ^3^, at 100:1 lipid to protein ratio (w/w). The detergent was removed using Bio-Beads SM-2 and the channel-incorporated liposome suspension was collected by centrifugation (60 minutes at 300,000 x g) and re-suspended in 10 mM HEPES (pH 7), 100 mM KCl. Samples were stored at -80 ºC.

**Pyrene-labeling of L90C KcsA mutant channel**

For an independent determination of the overall rotational correlation time of the detergent-solubilized KcsA channel the L90C mutant was used in order to chemically modify the cysteine residues with the long-lifetime fluorescent probe *N*-(1-pyrene) maleimide.^4^ After purification by affinity chromatography, the protein sample solubilized with 5 mM DDM in 20 mM Hepes, pH 7 buffer containing 100 mM KCl was first incubated with a 10-fold excess of the reducing agent TCEP dissolved in the same buffer. After reducing all cysteine residues, a 20-fold excess of *N*-(1-pyrene) maleimide in DMSO was added to the protein (the final volume of organic solvent was <0.5% of the total sample volume). This mixture was incubated for 3-4 hours at room temperature with agitation. The excess of dye was removed by subsequent binding of the protein to the Ni^2+^-Sepharose resin followed by extensive washing. Labelled (and unlabeled) protein was then eluted with 500 mM imidazole (as in a regular purification process) and dialyzed against the appropriate buffer. The labelling ratio (pyrene:KcsA tetramer) was determined by spectrophotometric quantification of the dye (ε_339nm_=40,000 M^-1^cm^-1^, ε_280nm_=20,000 M^-1^cm^-1^). This parameter ranged between 0.3 and 0.5 dye:tetramer, thus minimizing the possibility of pyrene excimer formation.^5^

**Electrophysiological** **recordings**

Inside-out patch clamp recordings were conducted on excised patches from asolectin giant liposomes containing W67 or W67 E71A KcsA as reported previously, ^6^ using an automated patch clamp system (Nanion Technologies, Germany) equipped with an external perfusion device. Gigaseals were obtained on NPC-1 borosilicate glass chips (Nanion Technologies, Germany) with resistances of 3-5 MΩ. After a stable seal was formed, the remaining liposomes were washed away with the corresponding intracellular buffer. Currents were recorded using an EPC-10 amplifier (HEKA Electronic, Lambrecht/Pfalzt, Germany), at a gain of 50 mV/pA. Data were digitized at a sampling rate of 40 kHz and low-pass filtered to 8 kHz (Bessel filter, HEKA amplifier). Afterwards, they were analyzed with Clampfit 10.3 (Molecular Devices, Axon Instruments). All measurements were taken at room temperature, with the extracellular solution containing 10 mM HEPES buffer (pH 7) and the intracellular solution 10 mM MES buffer (pH 4), both with 100 mM KCl.

**Steady-state fluorescence spectroscopy**

Steady-state fluorescence measurements were performed on a Horiba Jobin Yvon Fluorolog-3-21 spectrofluorometer (New Jersey, USA) using 0.5-cm pathlength quartz cuvettes at room temperature. For the fluorescence emission spectra, the DDM-solubilized KcsA W67 and KcsA W67 E71A samples were excited at 295 nm and the emission was recorded between 302 and 410 nm in 1-nm increments using excitation and emission slits with a bandpass of 5 nm. An adequate blank correction was made for all the spectra. The results are expressed in terms of either the observed fluorescence intensity at a given wavelength or as the fluorescence spectral center of mass (intensity-weighted average emission wavelength, <λ>), as defined by Equation S1,

| $<\lambda>=\frac{\sum_{i} \lambda_{i}\times I_{i}}{\sum_{i} I_{i}}$ | ((S1) |
| --- | --- |

Here, *I*_i_ is the fluorescence intensity measured at a wavelength λ_i_.

Steady-state fluorescence anisotropy measurements, <*r*>, were calculated according to:

| $<r>=\frac{I_{\mathrm{VV}}-{GI}_{\mathrm{VH}}}{I_{\mathrm{VV}}+2 GI_{\mathrm{VH}}}$ | ((S2) |
| --- | --- |

where *I*_VV_ and *I*_VH_ are the fluorescence intensities (blank subtracted) of the vertically and horizontally polarized emission, when the sample is excited with vertically polarized light, respectively. The *G* factor (*G*= *I*_HV_/*I*_HH_) is an instrument correction factor which takes into account the transmission efficiency of the monochromator to the polarization state of the light. Since the steady-state anisotropy values of tryptophan are strongly dependent on the excitation wavelength, ^7^ this parameter was routinely measured at 340 nm using an excitation wavelength of 300 nm to maximize the dynamic range of the time-resolved anisotropy measurements. Ten measurements were done for each sample and at least three independent samples were used to calculate average steady-state anisotropy values (± standard deviation, S.D.).

The Förster radius, *R*_o_, or the critical distance at which the energy transfer efficiency is 50% for an isolated donor–acceptor pair, was calculated for the W67 residues in the detergent-solubilized quadruple mutant channel using the following relationship:^8^

| $Ro (Å)={0.2108 [\kappa^{2}n^{-4} {}_{D} J\left( \lambda\right)]}^{\frac{1}{6}}$ | ((S3) |
| --- | --- |

where the orientation factor, ^2^, and the refractive index of the medium, *n*, were assumed to be 2/3 (i.e. the dynamical isotropic limit value ^9^) and 1.6 ^10^, respectively. In this equation, using nm units for the calculation of the overlap integral, *R*_o_ is obtained in Å. The donor quantum yield, Φ_D_, for the single tryptophan residue in each subunit of the detergent-solubilized KcsA W67 channel was determined to be 0.29 in the presence of 200 mM KCl using a reference solution of *N*-acetyl-*L-*tryptophanamide (NATA) in water (Φ= 0.14)^11^ . The spectral overlap integral, *J*(λ) was calculated using the absorption and normalized fluorescence emission spectra of W67KcsA in 20 mM Hepes, 5 mM DDM, 100 mM KCl, pH 7 buffer (*J*= 1.14 M^-1^cm^-1^nm^4^).

**Time-resolved fluorescence spectroscopy**

Time-resolved fluorescence and anisotropy measurements with picosecond resolution were obtained using the single photon timing (SPT) technique, as described elsewhere.^12^ The fluorescence decays of the 6 μM detergent-solubilized W67 KcsA samples (λ_exc_= 300 nm) and pyrene-labeled L90C KcsA channels (λ_exc_= 335 nm) were measured at 345 nm and 400 nm, respectively, using an emission polarizer set at the magic angle (54.7^o^) relative to the vertically polarized excitation beam produced by a frequency doubled Rhodamine 6G laser, as previously described.^13^ In both cases, the emission wavelength was selected by a Jobin Yvon HR320 monochromator (Horiba Jobin Ivon Inc.). Adequate blank decays were also acquired but their photon counts were found to be negligible. The instrument response function (IRF) was recorded as excitation light scattered by a Ludox solution (silica, colloidal water solution, Aldrich, Milwaukee, WI). The data were collected using a multichannel analyzer with a time window of 1024 channels, at typically 39 and 71 ps per channel for the W67 KcsA and pyrene-labeled L90C KcsA mutant proteins, respectively. Usually, up to 50,000 and 20,000 counts were acquired in the peak channel of the IRF and decay curves, respectively. For measuring the time-resolved anisotropy decays, the parallel and perpendicular polarized components of the fluorescence (*I*_VV_(t) and *I*_VH_(t), respectively) to the vertical plane of polarization of the excitation beam were alternately recorded using the set of conditions previously described.

The fluorescence intensity decays, $I\left( t \right)$, were described by a sum of exponentials:

| $I\left( t \right)= \sum_{i=1}^{n} {}_{i} exp({-t}/{{}_{i}})$ | (S4) |
| --- | --- |

where ${}_{i}$ and ${}_{i}$ are the normalized amplitude and the lifetime of the $i$^th^ decay component, respectively. The amplitude-weighted average lifetime, ${<\tau>}_{1}$, is defined by:

| ${<\tau>}_{1}= \sum_{i=1}^{n} {}_{i}{}_{i}$ | (S5) |
| --- | --- |

The anisotropy decays, $r\left( t \right)$, were described by:

| $r\left( t \right)= \sum_{i=1}^{n} {}_{i}\exp\left( {-t}/{{}_{i}} \right)$ | (S6) |
| --- | --- |

where ${}_{i}$ and ${}_{i}$ are the amplitude and the rotational correlation time of the $i$^th^ decay component of the anisotropy, respectively.

Analysis of the fluorescence intensity decays was performed with the TRFA software (Scientific Software Technologies Center, Minsk, Belarus) which uses a non-linear least squares regression method based on the Levenberg– Marquardt algorithm. The anisotropy decays of the detergent-solubilized W67 and W67 E71A KcsA samples were analyzed as detailed in the Theory section. The time-resolved anisotropy measurements of the pyrene-labeled L90C KcsA channels solubilized in the detergent micelles were globally analyzed using a two-step procedure using the TRFA software as previously described.^12^ The usual statistical criteria, namely a reduced χ^2^ < 1.2 and a random distribution of weighted residuals and autocorrelation plots, were used to evaluate the goodness of the fits.^13^

**THEORY: Time-resolved anisotropy and homo-FRET in the tetrameric W67 KcsA mutant channel**

The structure of the KcsA-DDM complex and in particular the relative positions of the four W67 residues in the tetrameric channel allows considering a square geometry for FRET, the distance between neighboring tryptophan fluorophores being *R* (Figure 3). Owing to identical environments, all four tryptophan residues are spectroscopically equivalent, implying the possibility of fully reversible FRET, i.e., pure homo-FRET.^9^ This process of electronic excitation energy hopping was modelled as follows: two different rate constants for FRET are used for neighboring and non-neighboring tryptophan residues, respectively *k*_1_ and *k*_2_, with

| $k_{i}=\frac{1}{}\left( \frac{3}{2}{}_{i}^{2} \right)\left( \frac{R_{0}}{R_{i}} \right)^{6} (i=1,2)$ | (S7) |
| --- | --- |

where *τ* is the fluorescence lifetime in the absence of FRET, *R*_0_ is the critical radius computed with an orientational factor *κ*^2^ = 2/3, $R_{1}=R$ (neighboring tryptophan residues) and $R_{2}=\sqrt{2}R$ (non-neighboring residues). Neglecting the intrinsic decay, which appears only as a multiplicative function, the survival probabilities of the excited fluorophores obey the master equation (see e.g. Berberan-Santos et al.,1999)^14^

| $\frac{d}{dt}=$ | (S8) |
| --- | --- |

In this case,

| $=\left[ \begin{matrix} -\left( 2k_{1}+k_{2} \right) & k_{1} & k_{2} & k_{1} \\ k_{1} & -\left( 2k_{1}+k_{2} \right) & k_{1} & k_{2} \\ k_{2} & k_{1} & -\left( 2k_{1}+k_{2} \right) & k_{1} \\ k_{1} & k_{2} & k_{1} & -\left( 2k_{1}+k_{2} \right) \end{matrix} \right]$ | (S9) |
| --- | --- |

and

| $\left( t \right)=\left[ \begin{matrix} {}_{1}(t) \\ {}_{2}(t) \\ {}_{3}(t) \\ {}_{4}(t) \end{matrix} \right]$ | (S10) |
| --- | --- |

where *ρ_i_* (*i* = 1, 2, 3, 4) are the survival probabilities of the excited fluorophores. The eigenvalues of matrix **K** are $-2\left( k_{1}+k_{2} \right)$ (double degenerate), $-4k_{1}$ and 0. The solution of the master equation is

| $\left( t \right)=exp(t)\left( 0 \right)$ | (S11) |
| --- | --- |

with

| $exp(t)=\frac{1}{4}\left[ \begin{matrix} f_{1}(t) & f_{2}(t) & f_{3}(t) & f_{2}(t) \\ f_{2}(t) & f_{1}(t) & f_{2}(t) & f_{3}(t) \\ f_{3}(t) & f_{2}(t) & f_{1}(t) & f_{2}(t) \\ f_{2}(t) & f_{3}(t) & f_{2}(t) & f_{1}(t) \end{matrix} \right]$ | (S12) |
| --- | --- |

where

| $f_{1}\left( t \right)=1+2 \exp\left[ -2\left( k_{1}+k_{2} \right)t \right]+\exp\left( -4k_{1}t \right)$  $f_{2}\left( t \right)=1-\exp\left( -4k_{1}t \right)$  $f_{3}\left( t \right)=1-2 \exp\left[ -2\left( k_{1}+k_{2} \right)t \right]+\exp\left( -4k_{1}t \right)$ | (S13) |
| --- | --- |

Considering, without loss of generality, that the chromophore labelled as 1 is the initially excited one,

| $\left( 0 \right)=\left[ \begin{matrix} 1 \\ 0 \\ 0 \\ 0 \end{matrix} \right]$ | (S14) |
| --- | --- |

it follows from the above equations that

| $\left( 0 \right)=\frac{1}{4}\left[ \begin{matrix} f_{1}(t) \\ f_{2}(t) \\ f_{3}(t) \\ f_{2}(t) \end{matrix} \right]$ | (S15) |
| --- | --- |

The time-resolved fluorescence anisotropy, $r\left( t \right)$, of the system will then be described by

| $r\left( t \right)={}_{1}\left( t \right) r_{1}+{}_{2}\left( t \right) r_{2}+{}_{3}\left( t \right) r_{3}+{}_{4}\left( t \right)r_{4}$ | (S16) |
| --- | --- |

where $r_{1}$ is the anisotropy of the initially excited fluorophore and $r_{2}$, $r_{3}$ and $r_{4}$ are the anisotropies of the indirectly excited ones, respectively. In the absence of any local rotational dynamics, $r_{1}$ equals the fundamental anisotropy, $r_{0}$, of the fluorophore and $r_{2}=r_{4}=d_{1}r_{0}$ and $r_{3}=d_{2}r_{0}$ where *d*_1_ and *d*_2_ are the depolarization factors for nearest neighbors and for the tryptophan residue in the opposite vertex, respectively. The depolarization factors are given by:^9^

| $d_{i}=\frac{3\left\langle\cos^{2} {}_{i} \right\rangle-1}{2} (i=1,2)$ | (S17) |
| --- | --- |

where *α*_i_ is the angle between the emission transition dipoles of the initially excited tryptophan (donor) and a given acceptor (*i* = 1 for nearest neighbors, *i* = 2 for the non-nearest neighbor) and <…> represents an average over conformations. In this way, the fluorescence anisotropy, *r*(*t*), is

| $r\left( t \right)=\frac{r_{0}}{4}\left[ f_{1}\left( t \right)+2d_{1}f_{2}(t)+d_{2}f_{3}(t) \right]$ | (S18) |
| --- | --- |

The equation can then be rewritten as

| $r\left( t \right)=\frac{r_{0}}{4}\left[ \left( 1+2d_{1}+d_{2} \right)+2\left( 1-d_{2} \right)\exp\left[ -2\left( k_{1}+k_{2} \right)t \right]+\left( 1-2d_{1}+d_{2} \right)\exp\left( -4k_{1}t \right) \right]$ | (S19) |
| --- | --- |

It is interesting to note that the contribution of direct transfer between the fluorophores located at opposite vertices of the square is minor and mainly affects the long-time behavior of the anisotropy decay. Indeed, neglect of this contribution only modifies the double degenerate eigenvalue of matrix **K** from $-2\left( k_{1}+k_{2} \right)$ to $-2k_{1}$.

In fluid solution, an additional depolarization mechanism is the rotational motion of the KcsA-DDM complex as a whole. Assuming a single rotational correlation time for this motion, ${}_{g}$, the anisotropy decay becomes

| $r\left( t \right)=\frac{r_{0}}{4}\left[ \left( 1+2d_{1}+d_{2} \right)+2\left( 1-d_{2} \right)\exp\left[ -2\left( k_{1}+k_{2} \right)t \right]+\left( 1-2d_{1}+d_{2} \right)\exp\left( -4k_{1}t \right) \right] \cdot exp\left( {-t}/{{}_{g}} \right)$ | (S20) |
| --- | --- |

The general solution for the anisotropy decay of our system with a square geometry is therefore a function of six parameters: *r*_0_, *k*_1_, *k*_2_, *d*_1_, *d*_2_, and ${}_{g}$. The determination of *k*_1_, in particular, allows obtaining the nearest neighbor distance, *R*, if the respective orientational factor is known (Eq. S7).

A simplified model for homo-FRET was further considered by assuming that FRET is isotropic, i.e., there is no orientational dependence and the effective orientational factor, κ^2^ ≈ 2/3. In such a case,

| $k_{1}=\frac{1}{}\left( \frac{R_{0}}{R} \right)^{6}$ | (S21) |
| --- | --- |

and *k*_2_ = *k*_1_/8.

In addition, since the W67 residues have mixed polarization and some local rotational dynamics that helps depolarizing the emitted fluorescence ($r\left( 0 \right)$< $r_{0}$), it is reasonable to assume that the anisotropies of the indirectly excited fluorophores are zero ($r_{2}=r_{3}=r_{4}=0)$. Eq. S20 can thus be re-written as

| $r\left( t \right)=\frac{r\left( 0 \right)}{4}\left[ 1+\exp\left( -4k_{1}t \right)+2\exp\left( -\frac{9}{4}k_{1}t \right) \right] \cdot exp\left( {-t}/{{}_{g}} \right)$ | (S22) |
| --- | --- |

The anisotropy decays obtained for the detergent-solubilized W67 and W67 E71A KcsA mutant channels were fitted with Eq. S22 using a homemade software that uses a non-linear least-squares reconvolution method based on the Levenberg– Marquardt algorithm. This is important for the determination of short depolarizing times. Only two fitting parameters, $r\left( 0 \right)$ and$k_{1}$, were used; the global rotational correlation time, ${}_{g}$, was determined in independent experiments using the pyrene-labeled L90C KcsA mutant solubilized in 5 mM DDM and kept fixed during these analyses. The inter-tryptophan lateral distance *R* can be directly calculated via *k*_1_ (Eq. S21); since the W67 residues present a complex fluorescence intensity decay, their intensity-weighted mean fluorescence lifetime, ${<>}_{2}$, was used in these calculations instead of τ:

| ${<\tau>}_{2}= \sum_{i=1}^{n} f_{i}{}_{i}$ | (S23) |
| --- | --- |

where $f_{i}$ is the fractional fluorescence intensity emitted by the *i*^th^ fluorescence decay component*,*

| $f_{i}=\frac{{}_{i}{}_{i}}{\sum_{i=1}^{n} {}_{i}{}_{i}}$ | (S24) |
| --- | --- |

The steady-state anisotropy can be calculated from an average of the anisotropy decay, *r*(*t*), over the intensity decay, *I*(*t*) ^15^:

| $<r>=\frac{\int_{0}^{\infty} I\left( t \right)r\left( t \right)dt}{\int_{0}^{\infty} I\left( t \right) dt}$ | (S25) |
| --- | --- |

For a single-exponential intensity decay, substitution of Eqs.S4 and S22 into Eq. S25 yields:

| $<r>=\frac{r\left( 0 \right)}{4}\left[ \frac{1}{1+\tau/{{}_{g}}}+\frac{1}{1+\tau/{{}_{g}}+4\left( {R_{0}}/R \right)^{6}}+\frac{2}{1+\tau/{{}_{g}}+\frac{9}{4}\left( {R_{0}}/R \right)^{6}} \right]$ | (S26) |
| --- | --- |

It is important to consider two limiting cases: (*i*) when $R$ >> $R_{0}$ (i.e., in the absence of homo-FRET), Eq. S26 reduces to the well-known Perrin equation, and (*ii*) for ${}_{g}$ >> $\tau$, Eq. 26 simplifies to:

| $<r>=\frac{r\left( 0 \right)}{4}\left[ 1+\frac{1}{1+4\left( {R_{0}}/R \right)^{6}}+\frac{2}{1++\frac{9}{4}\left( {R_{0}}/R \right)^{6}} \right]$ | (S27) |
| --- | --- |

Figure S4 displays the steady-state emission anisotropy of a tetramer with a square geometry as a function of the relative lateral inter-fluorophore distance, $R/{R_{0}},$ in the absence of any significant rotational tumbling of the oligomer within the experimental time window of the fluorescence decay. Two useful regimes can be clearly identified: for $R$< 0.7$R_{0}$, the emission anisotropy allows evaluating the stoichiometry of the oligomer, as previously described by Runnels and Scarlata.^16^ In this case, homo-FRET among the fluorophores present in each oligomer is so efficient that the emission anisotropy becomes independent of *R*, reaching a plateau value of $r\left( 0 \right)$/4. On the other hand, for 0.8 < $R/{R_{0}}$ <1.7, the steady-state emission anisotropy becomes an extremely sensitive reporter of conformational changes within the tetramer that result in alterations of the lateral inter-fluorophore distance, *R*. Additionally, at large separation distances (i.e. for $R$> 1.8 $R_{0}$), fluorescence emission arises primarily from the initially excited monomer due to the absence of homo-FRET, and the emission anisotropy converges to that of an isolated monomer ( $r\left( 0 \right)=$ 0.35 in this case) . It should be noted that the curve simulated using Eq. S27 does not overlap with the Runnels and Scarlata prediction (calculated with Eq. 18 from their work^16^), because the authors in their mathematical treatment used the approximation that all molecules in a cluster of *N* molecules interacted equally (tetrahedron).

**Revisiting the assumptions of the homo-FRET model**

Considering the symmetric arrangement of the four channel subunits (four-fold axis of symmetry), it was possible to derive an analytical solution for the single tryptophan W67 KcsA channel undergoing homo-FRET within a square geometry. The time-resolved anisotropy measurements were analyzed according to this homo-FRET formalism by taking into account both the local and global reorientational motions of the fluorophores and DDM-KcsA complex, respectively. From the fitted lateral rate constants of homo-FRET$k_{1}$, the relative inter-subunit neighboring distances between the W67 residues in both mutant KcsA channels were calculated using the Förster formalism. The four main assumptions considered in the derivation of this model were: (**i**) pure homo-FRET, (**ii**) isotropic FRET, (**iii**) unpolarized emission of indirectly excited tryptophan residues, and (**iv**) isotropic rotational diffusion of the KcsA:DDM complex. These are now briefly discussed. (**i**) Pure homo-FRET means not only that FRET can take place between tryptophan residues, but also that it is fully reversible. This implies that all four fluorophores are spectroscopically equivalent, as expected in a tetrameric channel where the W67 residues have inner shielded locations with identical local environments. (**ii** and **iii**) The photophysics of tryptophan is noticeably complex: (a) the near UV electronic absorption of tryptophan has contributions from two low-lying singlet excited states which are designated ^1^L_a_ and ^1^L_b_, with nearly perpendicular transition moments.^7,17^ The decomposition of the tryptophan absorption spectrum into its ^1^L_a_ and ^1^L_b_ transitions at 77 K in a propylene glycol glass has been reported by Valeur and Weber.^7^ However, it is important to be aware that at room temperature there is some broadening of the ^1^L_a_ and ^1^L_b_ bands. Therefore, there may be significantly higher ^1^L_b_ absorption at 300 nm than would be predicted from the low-temperature spectral decomposition, as suggested by Fleming and collaborators ^18^. (b) There is an ultrafast internal conversion between the singlet excited states ^1^L_b_ and ^1^L_a_ (in the order of some fs ^19,20^, and therefore the emissive state of tryptophan residues in proteins is always the ^1^L_a_ state. To the best of our knowledge, there is only one reported exception to this rule ^21^. (c) The orientational dependence of FRET is expressed by the orientational factor *κ*^2^ that takes values between 0 and 4. Use of the average value, 2/3, is usually invoked with respect to the dynamic regime (fast isotropic rotational motion of the partners),^9^ which is not strictly the case here; however, mixed polarization in the acceptor absorption spectrum (^1^L_a_ and ^1^L_b_ transitions, with nearly orthogonal transition moments) is equivalent to rotational randomization of the orientations.^22^ In fact, the overlap between the emission and absorption bands of the single tryptophanyl residue in W67 KcsA occurs within the spectral region where there are pronounced variations in the fundamental anisotropy of tryptophan with the excitation wavelength used [280 – 300 nm], implying variable fractional contributions of each state to the absorption band (mixed polarization of the absorption spectrum of tryptophan). For example, the minimum anisotropy near 290 nm is due to a maximum in the absorption of the ^1^L_b_ state. ^7^ This means that homo-FRET between the tryptophanyl residues involves both ^1^L_a_ → ^1^L_a_ and ^1^L_a_ → ^1^L_b_ near orthogonal transfer processes. Therefore, energy transfer orientational requirements are relaxed and the situation approaches the dynamic regime (fast rotational motion) even when the relative orientational distribution is not completely random. (d) Finally, (fast) local internal reorientations of Trp residues (rapid vibrational (flipping) or rotational motions of Trp residues during their excited-state lifetime), can be inferred from the previously mentioned decrease observed in the initial anisotropy, *r*(0), of the detergent-solubilized W67KcsA mutant channel in relation to the expected value for its fundamental anisotropy, *r*_0_ (*r*(0)~ 0.21 < *r*_0_ (Trp) ~ 0.3 at λ_ex_= 300 nm).^7^ These will also contribute to reduce the orientational constraints. Thus, between the events of excitation of a tryptophan residue and the emission of light by fluorescence by a different one, several depolarizing steps contribute to the randomization of the orientations of the interacting dipoles, warranting the approximations used in our model that κ^2^= 2/3 and the anisotropy of the indirect excited tryptophanyl residues is 0 (*r*_2_= *r*_3_= *r*_4_= 0). (**iv**) Finally, although the detergent-solubilized W67 mutant channel is not strictly spherical, its rotational motion in buffer, as described by the fluorescence anisotropy of the long-lived attached fluorescent probe (pyrene), is dominated by a long rotational correlation time of ~ 43 ns, which was found to be essentially independent of the experimental conditions used (pH and ionic composition of the buffer), and consequently was subsequently fixed in all homo-FRET analyses.

The assumptions made can also be justified *a posteriori* from the fact that fitting the anisotropy decays with equation S22, using only two fitting parameters, is always satisfactory and yields consistent results. Eq. S20 implies, in general, different pre-exponential coefficients for a satisfactory fit. In particular, it follows from equation S22 that the fluorescence anisotropy decays, in the absence of rotational motion of the tetramer, converges to r(0)/4, as found experimentally at high potassium concentration, a result that immediately implies a negligible contribution from indirectly excited chromophores.

**Determination of the rotational correlation time of the KcsA-DDM complex**

In order to minimize the number of free fitting parameters in the analyses of time-resolved anisotropy decays, we sought to determine independently the rotational correlation time of the detergent-solubilized mutant channel, ${}_{g}$ (Eq.1). Accordingly, we decided to label the L90C mutant of KcsA with the *N*-1-pyrene maleimide dye (Figure S3A).

L90C KcsA is a well-characterized mutant of this channel which has already been used in both X-ray crystallography and fluorescence studies. ^23,24^ The pyrene derivative was used as an extrinsic fluorophore since it has a much longer fluorescence lifetime ^4^ than the Trp residues of W67 KcsA mutant protein, providing an adequate time window to measure with accuracy the rotational correlation time of the mutant protein-DDM complex. The dye:KcsA (tetramer) ratio obtained for the pyrene-labeled L90C KcsA mutant was 0.3-0.5, excluding the possibility of pyrene excimer formation.^5^ The fluorescence intensity decays of the pyrene-labelled mutant solubilized in 5 mM DDM were measured at both pH 7 (closed state) and 4 (open state) and in the presence of 200 mM K^+^ or Na^+^ (Figure S3B), since these correspond to the extreme conformations of the selectivity filter under study.^23,25^ As expected, the intensity-weighted mean fluorescence lifetime of the detergent-solubilized pyrene-labeled protein was very long (<τ>_2_~ 70 – 80 ns (Table S3). In addition, the anisotropy decays of this pyrene-conjugated mutant were found to be essentially independent of the experimental conditions used (monovalent cation/pH) (Figure S3C). These decays were analyzed using a two-step procedure: first, they were individually fitted to a sum of three exponentials (Table S4): the shorter correlation times (${}_{1}$ ~ 0.2 and ${}_{2}\sim$ 7 ns, respectively) were ascribed to the internal mobility of the covalently-bound dye and the dominant long rotational correlation time, ${}_{3}$~ 43 +/- 3 ns, was assigned to the overall tumbling of the tetrameric KcsA-DDM complex in buffer solution. Remarkably, this parameter was found to be essentially independent of the experimental conditions tested and therefore was kept fixed in a subsequent global analysis of the decay curves using four exponentials. This procedure only slightly increased the quality of the fits by taking into account the contribution of a residual long correlation time, ${}_{4}$~ 100 - 300 ns (β_4_~0.02), which was assigned to the formation of large supramolecular assemblies (which could only be detected using this long-lived fluorescent probe), as already described in a previous analytical ultracentrifugation study (data not shown).^26^ Previous studies performed by analytical ultracentrifugation, SAXS or NMR estimated a molecular weight of ~150 kDa for KcsA complexed to DDM, sodium dodecyl sulfate (SDS), or fos-choline detergents, but their rotational correlation time was only determined in the last two works, with values around 36 and 60 ns, respectively ^27,28^, which are close to our measurements. According to these time-resolved results, all subsequent analyses of the anisotropy decays obtained for the W67 KcsA channel were performed using a fixed value of ${}_{g}$= 43 ns.

**SUPPLEMENTARY FIGURES**

**Figure S1**

**
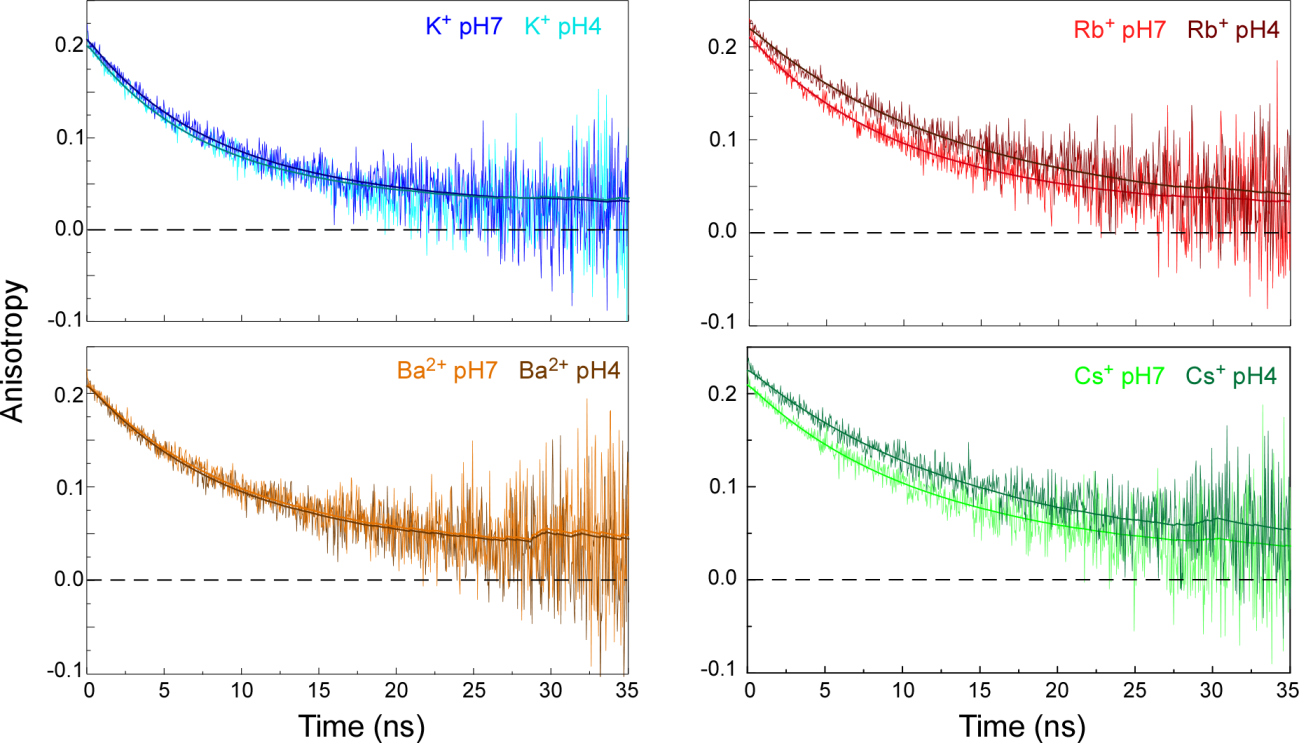
**

**Figure S1.** **Influence of pH-induced channel gating on the fluorescence anisotropy decays of the detergent-solubilized W67 KcsA channel.** Representative fluorescence anisotropy decays (λ_ex_= 300 nm; λ_em_= 345 nm) of 6 µM W67 KcsA in detergent micelles obtained at pH 7 (closed state) and pH 4 (open state) in the presence of 200 mM K^+^, Rb^+^ and Cs^+^ and 10 mM Ba^2+^. Buffer exchange from pH 7 to pH 4 results in a slower anisotropy decay in the presence of the cations that do not bind to the S2 position of the SF (Rb^+^ and Cs^+^), but not K^+^ nor Ba^2+^, which in fact can interact with that site (among others)^16^. The solid lines are the best fit of Eq. 1 to *r*(*t*) with ${}_{g}$= 43 ns kept as a fixed parameter in the analyses.

**Figure S2**

**
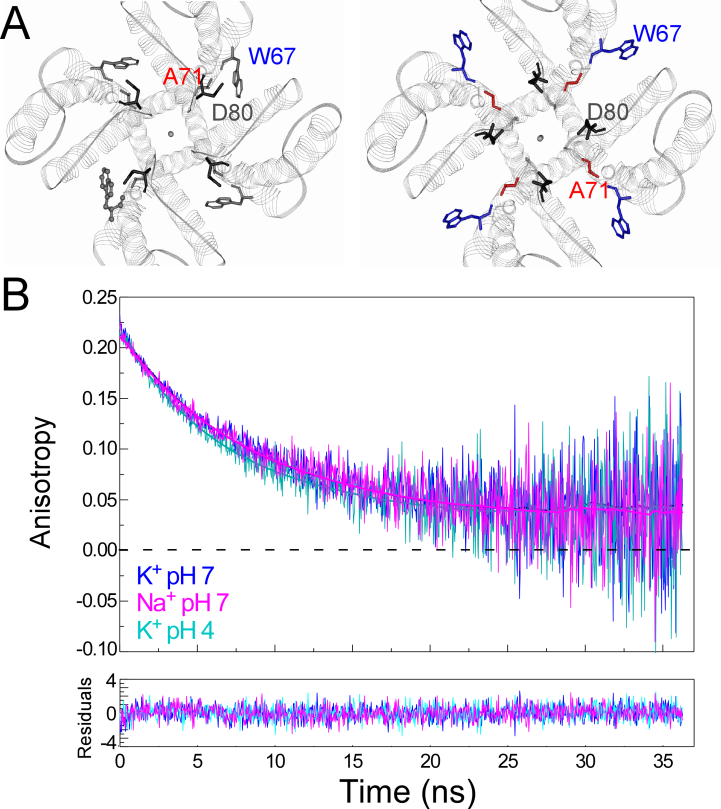
**

**Figure S2.** **Influence of ion species and pH-induced channel gating on the fluorescence anisotropy decays of the detergent-solubilized W67 E71A KcsA channel. (A)** The top panel presents a top view of the crystallographic structure of E71A KcsA, where two distinct W67 rotamers were observed according to the non-flipped (*left*, PDB ID: 1ZWI) or flipped (*right*, PDB ID: 2ATK) arrangement of D80 residue. **(B)** Representative fluorescence anisotropy decays of 6 µM W67 E71A KcsA in detergent micelles obtained in the presence of 200 mM K^+^ or 1 M Na^+^, at pH 7 (closed state) and 200 mM K^+^ at pH 4 (open state), respectively (λ_ex_= 300 nm; λ_em_= 345 nm). The solid lines are the best fit of Eq. 1 to *r*(*t*) with *ϕ*_g_ = 43 ns kept as a fixed parameter in the analysis.

**Figure S3**


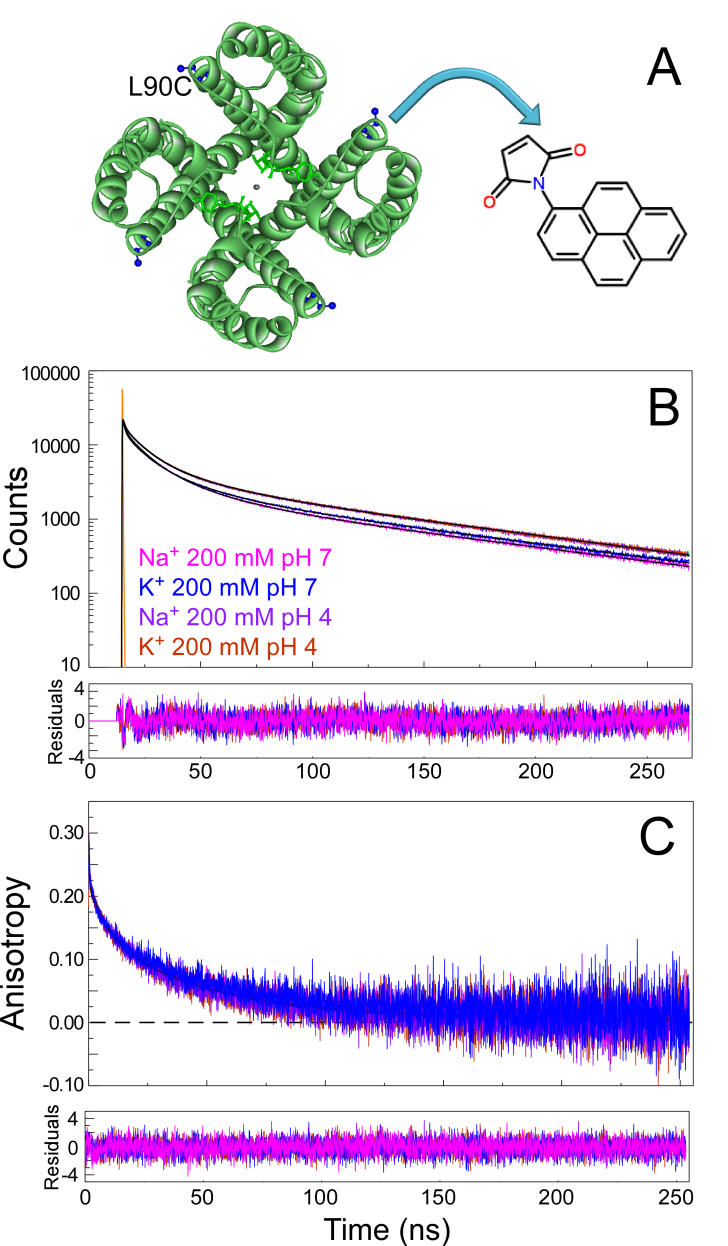


**Figure S3. Overall rotational tumbling of the W67 KcsA-DDM complex probed by time-resolved fluorescence anisotropy measurements. (A**) Top view of the KcsA tetramer (PDB ID: 1K4C) showing the location of the L90C mutation used to incorporate the *N*-1-pyrene maleimide fluorescent probe.(**B**) Representative fluorescence intensity decays, *I*(*t*), of 6 µM pyrene-labeled L90C KcsA mutant (tetramer:dye labeling ratio <1) in the presence of 20 mM Hepes, pH 7 buffer or 10 mM succinic acid, pH 4 buffer, including 5 mM DDM and 200 mM Na^+^ or K^+^ (λ_ex_= 335 nm; λ_em_= 400 nm). The solid lines are the best fit of Eq. S4 to the fluorescence decays. The IRF function is represented in yellow. (C) Fluorescence anisotropy decays of the same samples. The solid lines are the best fit of Eq. S6 to the anisotropy decays. The rotational dynamics of the detergent-solubilized pyrene labeled W67 KcsA was found to be essentially independent of the pH and ionic composition of the medium.

**Figure S4**


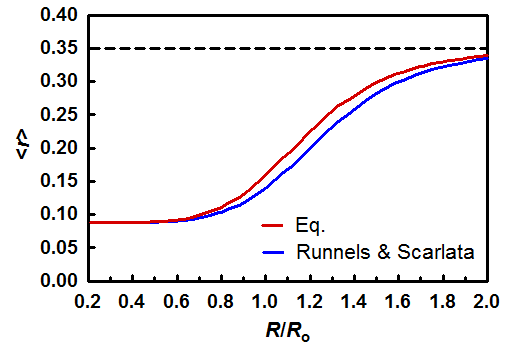


**Figure S4.** **Theoretical variation of the steady-state emission anisotropy of a tetramer as a function of the relative lateral inter-fluorophore distance, *R*/*R*_0_.** The red curve was calculated using Eq. S27 with $r\left( 0 \right)=$ 0.35 (square geometry) and the blue curve was obtained using Eq. 18 from the work of Runnels and Scarlata (*N*= 4, *r*_1_= 0.35 and *r*_et_= 0 ^16^ (tetrahedron geometry). The emission anisotropy of the tetrameric arrangement is extremely sensitive to the lateral inter-fluorophore distance for 0.8< *R*/*R*_0_ <1.7. For *R*< 0.7$R_{0}$, a plateau level of *r*(0)/4 is reached, i.e. the emission anisotropy reflects the stoichiometry of the oligomer.

**SUPPLEMENTARY TABLES**

**Table S1. Summary of the steady-state fluorescence parameters obtained at room temperature for the detergent-solubilized KcsA W67 and W67 E71A mutant channels at pH 7 and pH 4 and in the presence of 200 mM of each salt (except when noted).**

| **KcsA**  **mutant** | **Salt** | **<λ>, nm**  **pH 7** | **<λ>, nm**  **pH 4** | **Δ<λ>** | **<*r*>pH 7** | **<*r*>**  **pH 4** | **Δ<*r*>** |
| --- | --- | --- | --- | --- | --- | --- | --- |
| W67 | TBA^+*^ | 336.3 ± 0.3 | 336.6 ± 0.1 | 0.3 | 0.180 ± 0.002 | 0.175 ± 0.001 | 0.006 |
|  | Ba^2+**^ | 335.3 ± 0.7 | 335.1 ± 0.3 | -0.2 | 0.139 ± 0.001 | 0.140 ± 0.001 | 0.001 |
|  | Na^+^ | 335.2 ± 0.2 | 335.9 ± 0.2 | 0.7 | 0.166 ± 0.004 | 0.175 ± 0.002 | 0.012 |
|  | Cs^+^ | 334.5 ± 0.3 | 335.8 ± 0.1 | 1.3 | 0.149 ± 0.003 | 0.164 ± 0.003 | 0.015 |
|  | Rb^+^ | 334.0 ± 0.2 | 334.9 ± 0.1 | 0.9 | 0.143 ± 0.004 | 0.158 ± 0.002 | 0.015 |
|  | K^+^ | 333.9 ± 0.1 | 334.3 ± 0.2 | 0.4 | 0.135 ± 0.004 | 0.139 ± 0.003 | 0.004 |
|  |  |  |  |  |  |  |  |
| W67 E71A | Na^+ ***^ | 333.9 ± 0.1 | - | - | 0.13 ± 0.001 | - | - |
|  | Cs^+^ | 333.9 ± 0.05 | 334.6 ± 0.2 | 0.7 | 0.128 ± 0.002 | 0.127 ± 0.001 | 0.001 |
|  | Rb^+^ | 333.7 ± 0.1 | 334.5 ± 0.2 | 0.8 | 0.128 ± 0.001 | 0.127 ± 0.001 | 0.001 |
|  | K^+^ | 333.6 ± 0.2 | 334.3 ± 0.3 | 0.7 | 0.132 ± 0.004 | 0.129 ± 0.002 | 0.003 |

Intensity-weighted average emission wavelength <λ> (λ_ex_= 295 nm) and steady-state anisotropy <*r*> (λ_ex_= 300 nm; λ_em_= 345 nm) were calculated using Eq. S1 and Eq.S2, respectively.

The calculated parameters represent the mean ± SD of at least three independent experiments

* The concentration of TBA^+^ used was 5 mM,

** The concentration of Ba^2+^ used was 5 mM

*** The concentration of Na^+^ used was 1 M.

**Table S2. Analysis of the fluorescence intensity decays obtained at room temperature for the detergent-solubilized KcsA W67 and W67 E71A mutant channels at pH 7 and pH 4 and in the presence of 200 mM of each salt (except when noted).**

| **KcsA**  **mutant** | **pH** | **Salt** | **α_1_** | **τ_1_**  **(ns)** | **α_2_** | **τ_2_**  **(ns)** | **α_3_** | **τ_3_**  **(ns)** | **<τ>_1_**  **(ns)** | **<τ>_2_**  **(ns)** |
| --- | --- | --- | --- | --- | --- | --- | --- | --- | --- | --- |
| W67 | 7 | TBA^+ *^ | 0.14 | 0.8 | 0.28 | 4.1 | 0.58 | 7.6 | 5.6 | 6.7 |
|  |  | Ba^2+^ | 0.13 | 0.7 | 0.25 | 3.7 | 0.61 | 6.6 | 5.1 | 6.0 |
|  |  | Na^+^ | 0.16 | 0.7 | 0.28 | 3.8 | 0.56 | 7.5 | 5.5 | 6.6 |
|  |  | Cs^+^ | 0.12 | 0.7 | 0.28 | 4 | 0.60 | 7.1 | 5.4 | 6.4 |
|  |  | Rb^+^ | 0.12 | 0.8 | 0.35 | 4.3 | 0.53 | 7.1 | 5.3 | 6.2 |
|  |  | K^+^ | 0.12 | 0.8 | 0.30 | 3.9 | 0.56 | 6.9 | 5.0 | 6.1 |
|  |  |  |  |  |  |  |  |  |  |  |
|  | 4 | TBA^+ *^ | 0.11 | 0.9 | 0.13 | 3.7 | 0.57 | 7.3 | 6.1 | 6.9 |
|  |  | Ba^2+^ | 0.13 | 0.8 | 0.24 | 4.1 | 0.63 | 6.9 | 5.4 | 6.3 |
|  |  | Na^+^ | 0.15 | 0.8 | 0.25 | 4.1 | 0.60 | 7.7 | 5.8 | 6.9 |
|  |  | Cs^+^ | 0.12 | 0.6 | 0.19 | 3.7 | 0.69 | 7.4 | 5.9 | 6.8 |
|  |  | Rb^+^ | 0.15 | 0.7 | 0.26 | 4.2 | 0.59 | 7.5 | 5.6 | 6.8 |
|  |  | K^+^ | 0.14 | 0.7 | 0.34 | 4.2 | 0.52 | 7.4 | 5.5 | 6.4 |
|  |  |  |  |  |  |  |  |  |  |  |
| W67 E71A | 7 | Na^+ **^ | 0.19 | 0.7 | 0.47 | 4.5 | 0.34 | 8.1 | 5.0 | 6.4 |
|  |  | Cs^+^ | 0.18 | 0.7 | 0.54 | 4.9 | 0.28 | 8.9 | 5.2 | 6.7 |
|  |  | Rb^+^ | 0.19 | 0.8 | 0.52 | 4.8 | 0.29 | 9.1 | 5.3 | 6.9 |
|  |  | K^+^ | 0.19 | 0.8 | 0.44 | 4.6 | 0.36 | 7.8 | 5.0 | 6.3 |
|  |  |  |  |  |  |  |  |  |  |  |
|  | 4 | Na^+^ | - | - | - | - | - | - | - |  |
|  |  | Cs^+^ | 0.16 | 1.0 | 0.63 | 5.3 | 0.20 | 9.1 | 5.4 | 6.5 |
|  |  | Rb^+^ | 0.16 | 1.0 | 0.63 | 5.2 | 0.22 | 8.9 | 5.4 | 6.4 |
|  |  | K^+^ | 0.13 | 0.9 | 0.65 | 5.3 | 0.22 | 8.8 | 5.5 | 6.4 |

α_i_ and τ_i_ are the normalized amplitude and the lifetime of the $i$ ^th^ decay component. The amplitude-weighted average fluorescence lifetime, <τ>_1_ and the average lifetime <τ>_2_, was calculated using Eq. S5 and 17, respectively (λ_ex_= 300 nm; λ_em_= 345 nm).

^*^  The concentration of TBA.Cl used was 5 mM,

^**^  The concentration of NaCl used was 1 M,

Typical errors of amplitudes: ~15-20%

Typical errors of individual lifetimes: ~5-10%

The calculated <τ>_1_ and <τ>_2_ represent the mean of at least three independent experiments

**Table S3. Analysis of representative fluorescence intensity decays obtained at room temperature for the detergent-solubilized pyrene-labeled L90C KcsA mutant channel at pH 7 and pH 4 and in the presence of the indicated salts at 200 mM.**  ${}_{i}$ and ${}_{i}$ are the normalized amplitude and the lifetime of the $i$ ^th^ decay component obtained from fitting Eq. S4 to the experimental data. The mean fluorescence lifetime, <τ>_1_, was calculated using Eq. S5 (λ_ex_= 335 nm; λ_em_= 400 nm).

| **pH** | **Salt** | **α_1_** | **τ_1_**  **(ns)** | **α_2_** | **τ_2_**  **(ns)** | **α_3_** | **τ_3_**  **(ns)** | **α_4_** | **τ_4_**  **(ns)** | **<τ>_1_**  **(ns)** | **<τ>2**  **(ns)** | **χ^2^** |
| --- | --- | --- | --- | --- | --- | --- | --- | --- | --- | --- | --- | --- |
| 7 | Na^+^ | 0.30 | 1.1 | 0.41 | 8.7 | 0.20 | 22.8 | 0.09 | 113.8 | 18.7 | 70.1 | 1.0 |
|  | K^+^ | 0.35 | 1.0 | 0.35 | 7.9 | 0.19 | 22.4 | 0.10 | 112.9 | 19.1 | 75.0 | 1.1 |
|  |  |  |  |  |  |  |  |  |  |  |  |  |
| 4 | Na^+^ | 0.33 | 1.0 | 0.32 | 8.9 | 0.20 | 24.2 | 0.15 | 111.8 | 24.7 | 81.4 | 1.1 |
|  | K^+^ | 0.27 | 1.0 | 0.40 | 8.2 | 0.19 | 22.9 | 0.13 | 110.8 | 22.1 | 76.5 | 1.1 |

**Table S4. Individual analysis of the fluorescence anisotropy decays obtained at room temperature for the detergent-solubilized pyrene-labeled L90C KcsA mutant channel at pH 7 and pH 4 and in the presence of 200 mM of the indicated salts.**  ${}_{i}$ and ${}_{i}$ are the amplitude and rotational correlation lifetime of the $i$ ^th^ decay component obtained from fitting Eq. S6 to the experimental data (λ_ex_= 335 nm; λ_em_= 400 nm).

| **pH** | **Salt** | **β_1_** | **φ_1_**  **(ns)** | **β_2_** | **φ_2_**  **(ns)** | **β_3_** | **φ_3_**  **(ns)** | **χ^2^** |
| --- | --- | --- | --- | --- | --- | --- | --- | --- |
| 7 | Na^+^ | 0.075 | 0.19 | 0.059 | 6.8 | 0.174 | 43.9 | 1.1 |
|  | K^+^ | 0.045 | 0.59 | 0.057 | 6.5 | 0.163 | 42.9 | 1.1 |
|  | | | | | | | | |
| 4 | Na^+^ | 0.164 | 0.07 | 0.056 | 6.8 | 0.174 | 38.4 | 1.0 |
|  | K^+^ | 0.046 | 0.29 | 0.053 | 9.0 | 0.160 | 44.3 | 1.1 |

**References**

1. Barrera, F. N. *et al.* Unfolding and refolding in vitro of a tetrameric, alpha-helical membrane protein: the prokaryotic potassium channel KcsA . *Biochem.*  **44,** 14344–14352 (2005).

2. Pace, C. N., Vajdos, F., Fee, L., Grimsley, G. & Gray, T. How to measure and predict the molar absorption coefficient of a protein. *Protein Sci.* **4,** 2411–2423 (1995).

3. Giudici, A. M. *et al.* Detergent-labile, supramolecular assemblies of KcsA: relative abundance and interactions involved . *Biochim.*  **1828,** 193–200 (2013).

4. Karpovich, D. S. & Blanchard, G. J. Relating the polarity-dependent fluorescence response of pyrene to vibronic coupling. Achieving a fundamental understanding of the py polarity scale. *J. Phys. Chem.* **99,** 3951–3958 (1995).

5. Strasburg, G. M., Leavis, P. C. & Gergely, J. Troponin-C-mediated calcium-sensitive changes in the conformation of troponin I detected by pyrene excimer fluorescence. *J. Biol. Chem.* **260,** 366–370 (1985).

6. Riquelme, G., Lopez, E., Garcia-Segura, L. M., Ferragut, J. A. & Gonzalez-Ros, J. M. Giant liposomes: a model system in which to obtain patch-clamp recordings of ionic channels . *Biochem.*  **29,** 11215–11222 (1990).

7. Valeur, B. & Weber, G. Resolution Of The Fluorescence Excitation Spectrum Of Indole Into The 1La And 1Lb Excitation Bands. *Photochem. Photobiol.* **25,** 441–444 (1977).

8. Berberan-Santos, M. N. & Prieto, M. J. E. Energy transfer in spherical geometry: Application to micelles. *J. Chem. Soc. Faraday Trans. 2 Mol. Chem. Phys.* **83,** 1391–1409 (1987).

9. Valeur, B. & Berberan-Santos, M. N. *Molecular Fluorescence*. (Wiley-VCH, 2012).

10. Babul, J. & Stellwagen, E. Measurement of protein concentration with interferences optics. *Anal. Biochem.* **28,** 216–221 (1969).

11. Stryer, L. Fluorescence Energy-Transfer As a Spectroscopic Ruler. *Annu. Rev. Biochem.* **47,** 819–846 (1978).

12. Melo, A. M., Fedorov, A., Prieto, M. & Coutinho, A. Exploring homo-FRET to quantify the oligomer stoichiometry of membrane-bound proteins involved in a cooperative partition equilibrium. *Phys. Chem. Chem. Phys.* **16,** 18105–18117 (2014).

13. Poveda, J. A., Prieto, M., Encinar, J. A., González-Ros, J. M. & Mateo, C. R. Intrinsic tyrosine fluorescence as a tool to study the interaction of the Shaker B ‘ball’ peptide with anionic membranes. *Biochemistry* **42,** 7124–7132 (2003).

14. Berberan-Santos, M. N., Choppinet, P., Fedorov, A., Jullien, L. & Valeur, B. Multichromophoric cyclodextrins. 6. Investigation of excitation energy hopping by Monte-Carlo simulations and time-resolved fluorescence anisotropy. *J. Am. Chem. Soc.* **121,** 2526–2533 (1999).

15. Lakowicz, J. R. *Principles of Fluorescence Spectroscopy.* . **3rd,** (Springer US , 2007).

16. Runnels, L. W. & Scarlata, S. F. Theory and application of fluorescence homotransfer to melittin oligomerization. *Biophys. J.* **69,** 1569–1583 (1995).

17. Callis, P. R. 1L(a) and1L(b) transitions of tryptophan: Applications of theory and experimental observations to fluorescence of proteins. *Methods in Enzymology* **278,** 113–150 (1997).

18. Hansen, J. E., Rosenthal, S. J. & Fleming, G. R. Subpicosecond fluorescence depolarization studies of tryptophan and tryptophanyl residues of proteins. *J. Phys. Chem.* **96,** 3034–3040 (1992).

19. Shen, X. & Knutson, J. R. Subpicosecond fluorescence spectra of tryptophan in water. *J. Phys. Chem. B* **105,** 6260–6265 (2001).

20. Bräm, O. *et al.* Relaxation dynamics of tryptophan in water: A UV fluorescence up-conversion and molecular dynamics study. *J. Phys. Chem. A* **114,** 9034–42 (2010).

21. Broos, J. *et al.* The emitting state of tryptophan in proteins with highly blue-shifted fluorescence. *Angew. Chemie - Int. Ed.* **46,** 5137–5139 (2007).

22. Haas, E., Ephraim-Katchalski-Katzir & Steinberg, I. Z. Effect of the Orientation of Donor and Acceptor on the Probability of Energy Transfer Involving Electronic Transitions of Mixed Polarization. *Biochemistry* **17,** 5064–5070 (1978).

23. Morais-Cabral, J. H., Zhou, Y. & MacKinnon, R. Energetic optimization of ion conduction rate by the K+ selectivity filter . *Nat.*  **414,** 37–42 (2001).

24. Blunck, R., Cordero-Morales, J. F., Cuello, L. G., Perozo, E. & Bezanilla, F. Detection of the opening of the bundle crossing in KcsA with fluorescence lifetime spectroscopy reveals the existence of two gates for ion conduction . *J.Gen.Physiol*  **128,** 569–581 (2006).

25. Lockless, S. W., Zhou, M. & MacKinnon, R. Structural and thermodynamic properties of selective ion binding in a K+ channel . *PLoS.Biol.*  **5,** e121 (2007).

26. Molina, M. L. *et al.* Clustering and coupled gating modulate the activity in KcsA, a potassium channel model . *J.Biol.Chem.*  **281,** 18837–18848 (2006).

27. Baker, K. A., Tzitzilonis, C., Kwiatkowski, W., Choe, S. & Riek, R. Conformational dynamics of the KcsA potassium channel governs gating properties . *Nat.Struct.Mol.Biol.*  **14,** 1089–1095 (2007).

28. Chill, J. H., Louis, J. M., Miller, C. & Bax, A. NMR study of the tetrameric KcsA potassium channel in detergent micelles . *Protein Sci.*  **15,** 684–698 (2006).
